# Effectiveness of Social Support for Community-Dwelling Elderly with Depression: A Systematic Review and Meta-Analysis

**DOI:** 10.3390/healthcare10091598

**Published:** 2022-08-23

**Authors:** Seon Heui Lee, Hanju Lee, Soyoung Yu

**Affiliations:** 1 Department of Nursing Science, College of Nursing, Gachon University, Incheon 21936, Korea; 2 Department of Nursing, Sangmyung University, Seoul 03016, Korea; 3 College of Nursing, CHA University, Pocheon 11160, Korea

**Keywords:** depression, aged, meta-analysis, systematic review

## Abstract

Depression in the elderly is an important health factor that requires intervention in the form of social support resources. The purpose of this study was to conduct a systematic review, while synthesizing available evidence on what kind of social support, such as social participation and social connection/network, is effective for depression in the elderly. We performed a quality assessment of the included studies using the revised Risk of Bias for Non-randomized Studies tool and a meta-analysis of studies published up to 14 May 2021. Of the 3449 studies, 52 were relevant to this study. The various types of social resource applications reported in these were classified into three types: social support, social participation, and social connection/network. The social support group had significantly lower depression compared to the control group (0.72 [0.65, 0.81], *p* < 0.00001, I^2^ = 92%). There was a significant decrease in depression in the social participation group compared to the control group (0.67 [0.56, 0.80], *p* < 0.00001, I^2^ = 93%) (2.77 [1.30, 5.91], *p* = 0.008, I^2^ = 97%) (0.67 [0.56, 0.80], *p* < 0.00001, I^2^ = 93%). Finally, the social connection/network group showed decreased depression compared to the control group (2.40 [1.89, 3.05], *p* < 0.00001, I^2^ = 24%) (0.83 [0.76, 0.90], *p* < 0.00001, I^2^ = 94%). The results of this systematic review confirmed the effects of various social support interventions in reducing depression among the elderly living in the community.

## 1. Introduction

The pathophysiological model of human health and disease has succeeded to some extent in identifying its causes and consequences through continuous research spanning decades. However, studies on the socio-psychological impacts on human health and disease have received relatively less attention [1]. Nevertheless, the social impact on health, especially the effect of social relations, has been reported through recent studies [2,3]. More specifically, the influence of social relationships is related to the number of human relations and their quality and effects in terms of social connections [1,4]. We need to pay attention to this social connection, especially in the case of the elderly, as it can be seen that the quantity and quality of social connections directly affect the health of the elderly [1,5,6]. For example, social isolation or loneliness in the elderly was found to be associated with a 50% increased risk of developing dementia [7], 30% increased risk of coronary artery disease or stroke [8], and 26% increase in mortality rates [9]. Although there are differences by country, 16.4% of the elderly living with their spouses and 21.7% of the elderly living with their adult children experienced depression, while 30.2% of the elderly living alone experienced depression [10]. Nurses, as health care professionals, need to identify the effects of social connections on diseases among the elderly and pay attention to depression in the elderly. In particular, among the suicide risk factors in the elderly, depression is an important factor that can be mediated and has been the subject of several suicide prevention studies [11]. However, late-life depression is not easy to detect and tends to be poorly treated due to the characteristics of masked depression and senile comorbidities [5,11]. Mental health deterioration, such as depression in old age, is related to the overall risk of life, and the urgency of intervention is emphasized [12]. As one of these interventions, research on social support and depression, including social relations, is being conducted. In a study on the relationship between social participation and depression risk among 4751 local residents over 60 years old, it was reported that the risk of depression symptoms in elderly people participating in social activities, volunteering, and donation decreased [13]. In a community-based random sampling study of 959 elderly people, in addition to other known causes, low social support was identified as the cause of depression. Appropriate social support has been reported to be important in alleviating pain caused by the loss of the elderly [14]. Another study identified the effect of social network composition on depression using panel data collected between 2005 and 2016 and explored how different social layers influence each other. A study has shown that community participation has a consistent advantage in reducing depression. In contrast, intimate partnerships have been reported to increase sensitivity to depression among the elderly by exposure to serious consequences of partner loss [6]. According to observational data of 6772 individuals from China’s health and retirement end study, elderly people in rural areas experience more severe depression than elderly people in urban areas, so an approach considering residential areas is needed rather than collective application of social support [15]. These studies emphasize that local and individual resources should be considered simultaneously and comprehensively to understand depression in old age [12]. Despite the growing discussion of social resources as non-material resources for depression in old age, another intervention study reported that social interactions among the elderly improved, but depressive symptoms did not decrease [11]. A systematic review reported in 2019 confirmed that appropriate and good social support reduces depression in the elderly [5] and reported that, when solving depression in an Asian context, it is necessary to integrate the designed programs and interventions of family institutions.

Based on these findings, a more robust systematic review is needed to summarize and synthesize evidence on the relationship between depression and social support and the progress of mental health problems related to depression in the elderly, and to confirm useful evidence for how, and in what context, the elderly can affect mental health recovery. Therefore, the purpose of this study is to conduct a systematic review of existing studies on depression in the elderly in the community, while synthesizing available evidence on what kind of social support, such as social participation and social connection/network, is most effective.

## 2. Materials and Methods

### 2.1. Search Strategy

This systematic review was conducted according to the Preferred Reporting Systems for Systematic Review and Meta-Analysis (PRISMA) guidelines. This study was conducted to investigate the risk factors of community-dwelling elderly people with depression. We searched relevant articles on 14 May 2021, using four databases: Ovid-Medline, Ovid-Embase, PsycINFO, and CINAHL. The search was performed using the terms that included Medical Subject Headings (MeSH). Keywords were as follows (aged OR older adults OR older persons OR old OR elderly) AND (independent living OR community dwelling OR community) AND (mental health OR depression OR emotional depression OR mood disorder OR affective disorder) AND (social support OR social network OR social relations OR tangible support OR social support network scale OR emotional support OR social support network) and combinations of these terms.

### 2.2. Study Selection

To rule out irrelevant studies, two reviewers independently reviewed the titles and abstracts of the articles, and a professional review was conducted of the relevant articles. The literature included in this systematic review was selected based on the following criteria: (1) English or Korean papers; (2) participants aged over 60 years; (3) community-dwelling elderly persons and not living in institutions; (4) participants with depression; (5) patients had social support; and (6) reports on predictive factors. The results of interest were predefined before conducting the review. Review articles, abstracts, conference posters, unpublished gray literature, protocols, not written in English or Korean, animal studies, and duplicate studies were excluded. Two authors checked reliability using Cohen’s kappa coefficient.

### 2.3. Data Extraction

Using pre-agreed data inclusion criteria, the two investigators independently extracted the data for this review. Disagreements were discussed among the reviewers until a consensus was reached. The following data were extracted from each article: author, year of publication, study design, country where study was conducted (city), object country, sample size, age, location, gender, social support measure, social support explanation, and depression measurement.

Social support is defined as the exchange of resources between at least two individuals, and one individual perceives it as promoting the welfare of the recipient [16]. High social support decreases depression. To measure social support, selected studies used the Oslo-3 Social Support Scale, using geriatric depression scale (GDS-15) [17,18] the Social Support Scale, using the Center for Epidemiologic Studies-Depression Scale (CES-D) [19] and the Chinese version of the Multidimensional Scale of Perceived Social Support [20]. In addition, the Positive Perceived Social Support [21] the Medical Outcome Study Social Support Survey [22] the Social Support Rating Scale [23] and the Social Support Index, which comprises both receiving and providing social support [24] were used to measure social support. Antonucci et al. [25] and Kim and Park [26] checked family and friend support, and Mechakra-Tahiri et al. [27] reported functional relationships, including social support and the presence of conflict.

Social participation is defined as the participation of individuals in social activities that allow interaction with others in the community [28]. One of the two measuring instruments indicates that high social participation decreases depression. Bai et al. [29] measured social participation using the framework of the World Bank’s Social Capital Assessment Tool, and Lee et al. [30] reported the number of social participations. Other instruments show that if there is a lot of social participation, the depression decreases [27,31,32]. Social networks are concepts related to the formal structure of social relations such as size, composition, frequency of contact, and boundaries [33]. Some social networks decrease depression, using the Lubben social network scale [34,35,36] and the framework of the World Bank’s Social Capital Assessment Tool [29]. Others decrease depression scores using the Revised Lubben Social Network Scale [37,38,39], the Chinese version of the Intergenerational Relationship Scale [20], the Social Support Scale [40], the version of portions of the social networks [25], network structure and social network function [19], network size and social interaction [30,41,42], and number of visitors per week [43].

### 2.4. Risk of Bias in Individual Studies

We performed a quality assessment of the included studies using the revised Risk of Bias for Non-randomized Studies (RoBANS) tool. There were eight domains of the tool, including the possibility of target group comparisons, target group selection, confounders, exposure measurement, blinding of assessors, outcome assessment, incomplete outcome data, and selective outcome reporting. Each domain was evaluated as “low”, “high”, or “unclear”. The outcome of the quality assessment was examined and agreed upon by the two reviewers.

### 2.5. Statistical Analysis

We conducted a meta-analysis using the Review Manager 5.4 (RevMan) program for the items that could be synthesized among the results of 52 included studies. The estimated effect, measured as odds ratio (OR) with 95% confidence intervals, was extracted. Between the studies, I^2^ statistics were used to evaluate statistical heterogeneity, and a fixed-effects model was used to analyze the data. We used a random effects model when heterogeneity was absent.

## 3. Results

### 3.1. Study Selection

**Figure 1:**
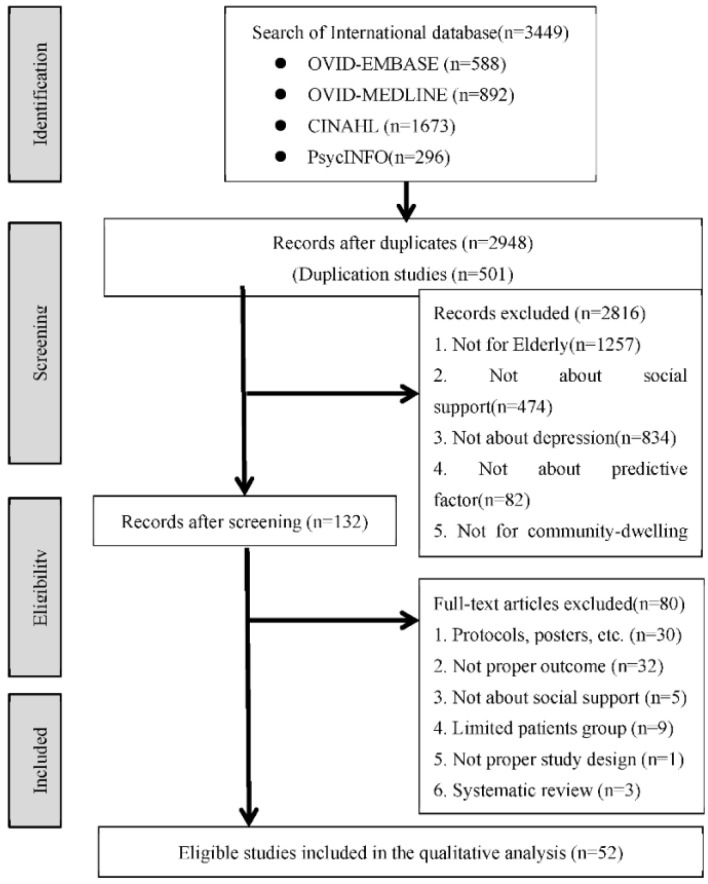
Flow chart.

After full-test reviews, 3449 studies were searched from the database. After excluding duplicates, 2948 studies remained. A full-text review revealed that 52 documents were relevant to this study. Figure 1 illustrates a flowchart of the study selection process. Reliability was checked by two reviewers using Cohen’s kappa coefficient (k = 0.85).

### 3.2. Study Characteristics

Table 1 represents the characteristics of the 52 included studies. The search was carried out on 14 May 2021. Studies published between 1997, when the search engine started, and 2021 were considered. The selected studies included 42 cross-sectional and 10 longitudinal studies. There were 37 studies from Asia, 11 from North America, two from Africa, one from Australia, and one from Europe. Specific items for each study were research design, sample size, age, gender of participants, depression and social support measurement tools, and other related variables. Supplementary Table S1 presents the characteristics of the subjects presented in each study, and Supplementary Table S2 presents various depression intervention methods, which are the main interests of this study, classified into social support, social participation, and social connection/network.

### 3.3. Risk of Bias within Studies

**Figure 2:**
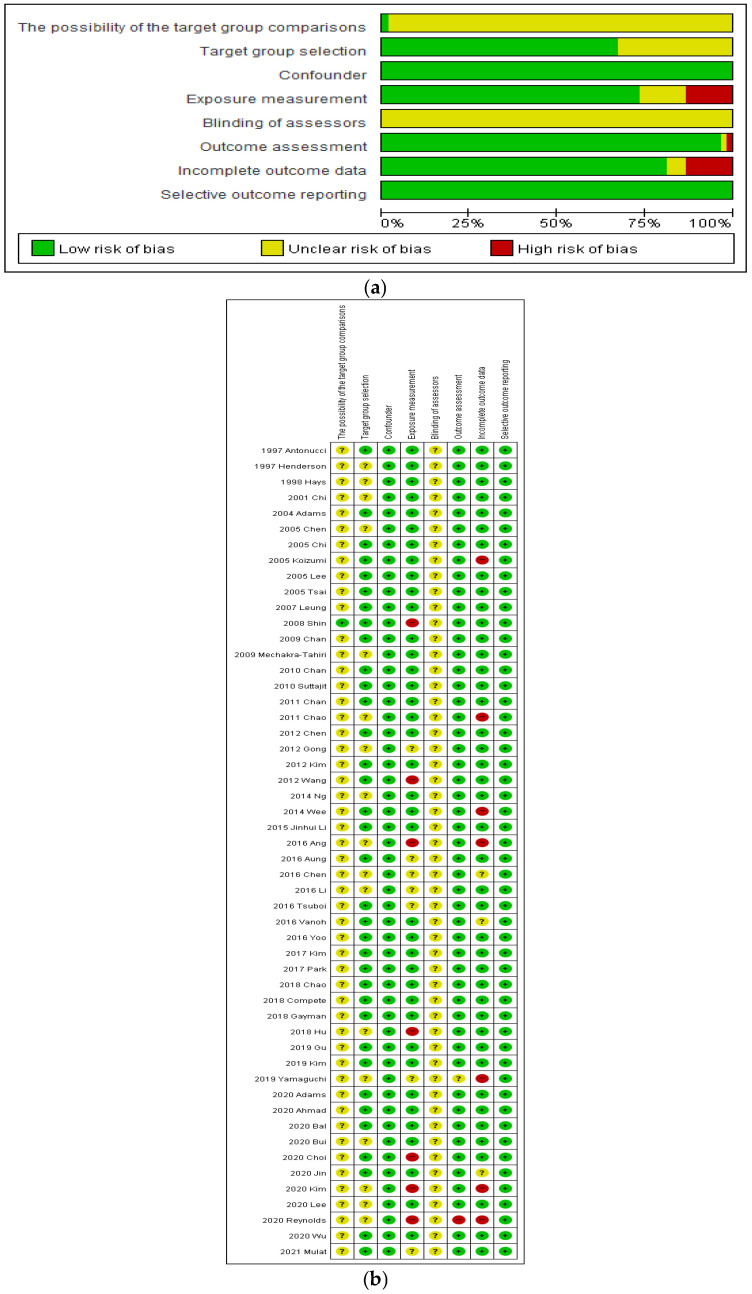
Quality assessment (**a**) Risk of bias graph; (**b**) Risk of bias summary.

The 15 selected studies were assessed using the revised (RoBANS) tool (Figure 2). The risk of possibility of target group comparisons and target group selection were low in 35 of the 52 studies. The risk of confounding and selective outcome reporting was low for all studies. The risk of exposure measurement was low in 39 studies. The risk of blinding of assessors was unclear. The risk of outcome assessment was low in 50 studies. The risk of incomplete outcome data was low in 42 studies.

### 3.4. Meta-Analysis of Selected Studies

#### 3.4.1. Social Support

**Figure 3:**
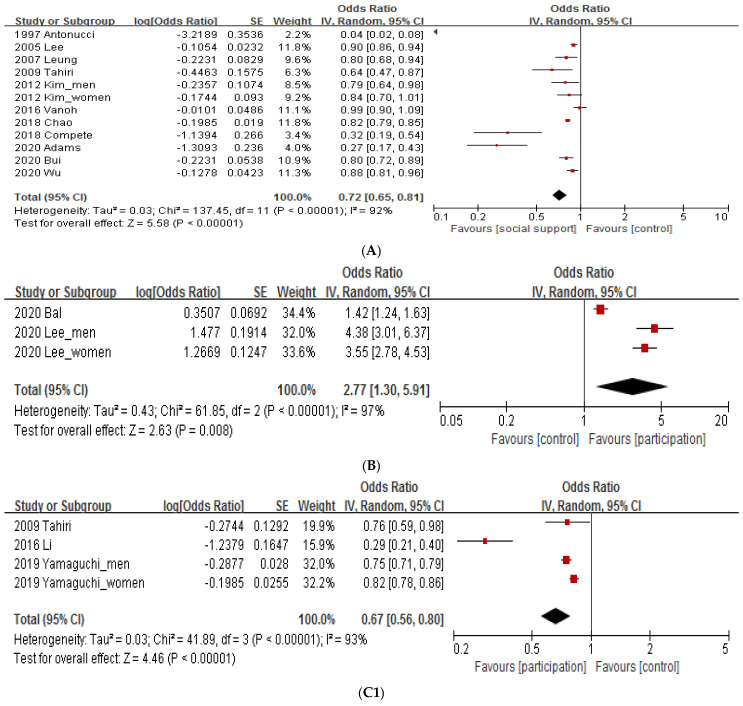

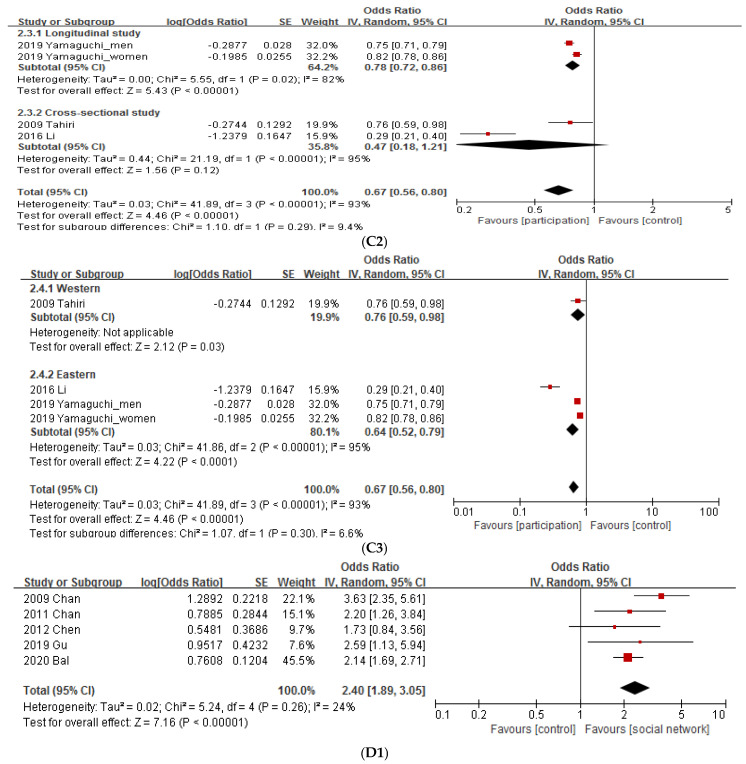

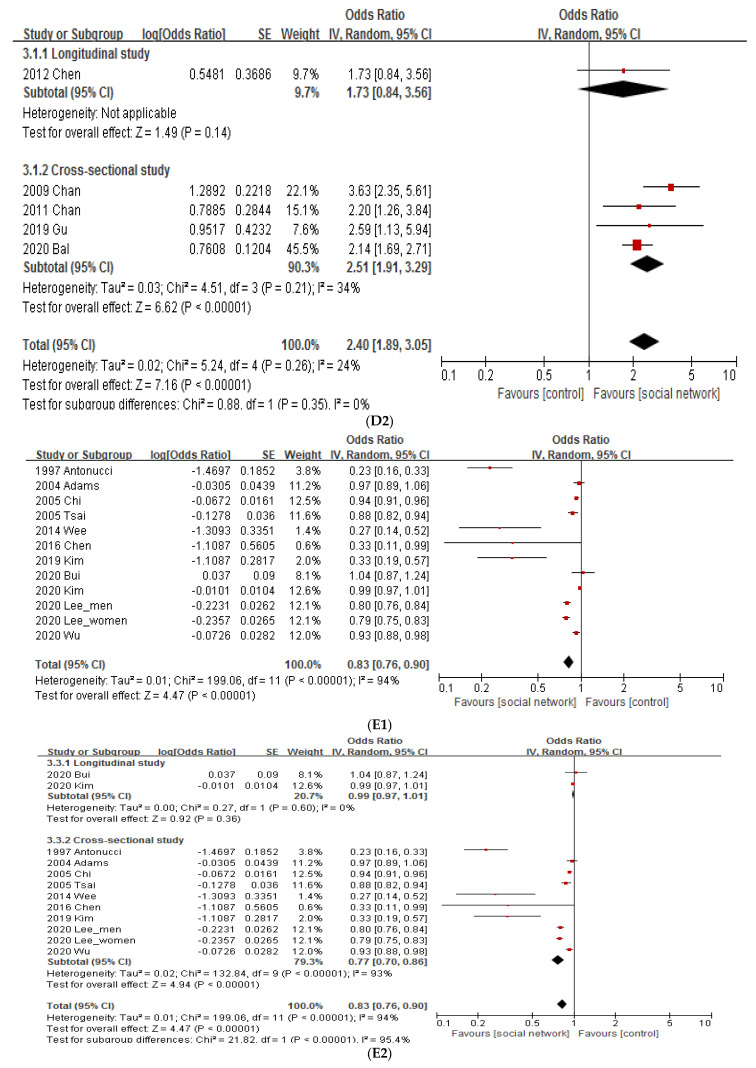

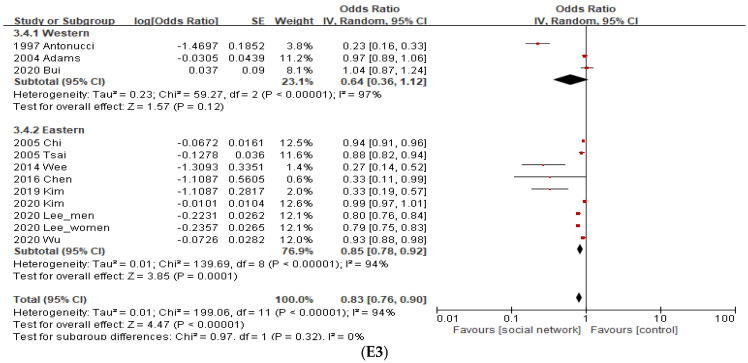
Meta-analysis. (**A**). Social support. (**B**) Social participation. (**C1**) Social participation. (**C2**). Subgroup analysis of social participation by study design. (**C3**). Subgroup analysis of social participation by published continents. (**D1**). Social network. (**D2**). Subgroup analysis of social network by study design. (**E1**). Social network. (**E2**). Subgroup analysis of social network by study design. (**E3**). Subgroup analysis of social network by published continents.

The social support group showed significantly decreased depression compared to the control group (0.72 [0.65, 0.81], *p* < 0.00001, I^2^ = 92%) (Figure 3A). Heterogeneity between studies was confirmed, and an additional subgroup analysis was performed. Subgroup analysis of social support by research type revealed that depression was significantly reduced in longitudinal studies (0.80 [0.72, 0.89], *p* < 0.0001) as well as in cross-sectional studies (0.71 [063, 0.80], *p* < 0.00001, I^2^ = 93%). Subgroup analysis was performed, but the heterogeneity was not reduced. In the subgroup analysis of social support of articles published by continents, social support significantly decreased depression compared to the control group in the western (0.45 [0.34, 0.61], *p* < 0.00001, I^2^ = 95%) and eastern continents (0.88 [0.83, 0.95], *p* = 0.0004, I^2^ = 54%). The heterogeneity value in eastern countries decreased, and depression decreased as social support increased in both groups.

#### 3.4.2. Social Participation

As shown in Figure 3B, high social participation increases depression scores, which means that depression decreases (for tools interpreting depression as decreasing as the number increases). The social participation group reported a significant decline in depression compared with the control group (2.77 [1.30, 5.91], *p* = 0.008, I^2^ = 97%), and all articles in the analyzed group were cross-sectional studies and the published continents were Eastern (Figure 3B).

Figure 3C1 shows that if there is a lot of social participation, the depression score decreases, which means that depression decreases. There was a significant decrease in depression in the social participation group compared to the control group (0.67 [0.56, 0.80], *p* < 0.00001, I^2^ = 93%) (Figure 3C1). Subgroup analysis was performed to reduce heterogeneity. In the subgroup analysis of social participation by research type, cross-sectional studies did not significantly reduce depression (0.47 [0.18, 1.21], *p* = 0.12, I^2^ = 95%), whereas longitudinal studies did (0.78 [0.72, 0.86], *p* < 0.00001, I^2^ = 82%) (Figure 3C2). In the subgroup analysis of social participation by published continents, depression in the social participation group was significantly reduced compared to the control group in the western (0.76 [0.59, 0.98], *p* = 0.03) and eastern continents (0.64 [0.52, 0.79], *p* < 0.0001, I^2^ = 95%) (Figure 3C3). The effect size of social participation in the eastern (0.64) was larger than that in the western continents (0.76).

#### 3.4.3. Social Connection and Social Network

As shown in Figure 3D1, many social networks increase depression scores. The social network group showed decreased depression compared with the control group (2.40 [1.89, 3.05], *p* < 0.00001, I^2^ = 24%), and all analyzed articles were published in Eastern continents (Figure 3D1). Subgroup analysis of social networks by published continents revealed that depression was significantly reduced in cross-sectional studies (2.51 [1.91, 3.29], *p* < 0.00001, I^2^ = 34%), but not in longitudinal studies (1.73 [0.84, 3.56], *p* = 0.14) (Figure 3D2).

Figure 3E1 shows that if there are many social networks, depression scores decrease. There was a significant decrease in depression in the social network group compared to the control group (0.83 [0.76, 0.90], *p* < 0.00001, I^2^ = 94%) (Figure 3E1). Subgroup analysis of social networks by research type revealed that depression was significantly reduced in cross-sectional studies (0.77 [0.70, 0.86], *p* < 0.00001, I^2^ = 93), but not in longitudinal studies (0.99 [0.97, 1.01], *p* = 0.36, I^2^ = 0%) (Figure 3E2). In the subgroup analysis of social networks by published continents, depression in the social network group was significantly reduced compared to the control group in eastern (0.85 [0.78, 0.92], *p* = 0.0001, I^2^ = 94%), but not in western continents (0.64 [0.36, 1.12], *p* = 0.12, I^2^ = 97%) (Figure 3E3).

## 4. Discussion

This study is the first to quantitatively synthesize the effect of social support on the elderly in the community using a meta-analysis. We conducted a systematic review of elderly depression in the community and conducted a meta-analysis on what kind of social resources among intervention methods such as social support, social participation, and social network are effective for elderly depression. As a result of the systematic review, which included a total of 52 studies, social support, social participation, and social connection/social network were identified as effective intervention methods for depression in the elderly in the community. In the subgroup analysis of social participation by research type, cross-sectional studies did not significantly reduce depression, whereas longitudinal studies did. Subgroup analysis of social networks by study type revealed that depression significantly decreased in cross-sectional studies but not in longitudinal studies. In the subgroup analysis of social networks by published continents, depression in the social network group was significantly reduced compared to the control group in the eastern, but not in the western continents.

### 4.1. Social Resources Were Effective Interventions for Depression in the Elderly of the Community

Social support, social participation, and social connection/network were identified as effective intervention methods for depression among the elderly in the community. It is necessary to discuss the results of this study and other research using a systematic review. According to a systematic review, which was already conducted 10 years ago, depression decreased in the group that applied social activities compared to the group that did not receive intervention [44]. However, since the number of trials was small, the results should be interpreted carefully. Nevertheless, the study showed that discussion groups sharing experiences with each other, designed to strengthen social networks and support, could help reduce loneliness among the elderly and increase social contact and social activities of the elderly. The 52 studies analyzed in this review also identified studies on interactions with families and neighbors as well as various programs; in this study, they were classified as social participation, including community and individual levels, and the results were found to have a positive effect on depression in the elderly. Another review paper analyzing 21 papers on depression intervention reported a study in which social support intervention had a small but significant effect on the reduction of depression symptoms, reporting that health manager and problem-solving interventions reduced depression [45]. As in the previous study, a systematic review in 2019 strongly supported the results of this study and confirmed the effect of depression intervention on 53 elderly residents in the community. The results of the study indicated that group-centered interventions and interventions including social factors have a positive effect on the mental health of participants. Therefore, group-based interventions should be considered first when considering that many elderly people have social difficulties [46]. Another recent systematic review found that through a total of 66 studies, social support has structural and functional aspects, and perceived social support is more generally measured than received social support. Social support has various factors depending on the study, but it was concluded that there is a clear association between social support and elderly depression [5]. Mohd et al. [5] classified social support into structural aspects such as marital status and residential environment and functional support such as emotional satisfaction. However, this study divided it into social support, social participation, and social connection/network. In conclusion, the results of the systematic review of the effects of depression intervention for the elderly consistently report that studies related to social support or participation are effective in depression.

### 4.2. Implications of the Meta-Analysis and Subgroup Analysis Results

We need to pay attention to the meta-analysis results of this study for the application of social support to practically reduce elderly depression. As mentioned in the results section, in this study, the effects were confirmed through meta-analysis by dividing the interventions for depression of the elderly into three categories: social support, social participation, and social connection/network. The overall meta-analysis results conducted in this study were found to indicate that social support interventions, including social support and social participation, help reduce depression. Additionally, the meta-analysis reported that heterogeneity between the studies was high; therefore, an additional subgroup analysis was performed.

The subgroup analysis of social participation intervention by type of study showed that cross-sectional studies did not significantly reduce depression, while longitudinal studies were found to reduce depression. In other words, long-term follow-up studies confirmed that social participation interventions were effective in depression, thus, they confirmed the positive effect of social participation on depression more clearly. This is because the descriptive cross-sectional study investigates at one point, so it is difficult to generalize the cause and result, while longitudinal research is a study that complements the disadvantages of cross-sectional research. Similarly, as a result of subgroup analysis by research type on social network intervention methods, depression did not decrease in cross-sectional studies, but significantly decreased in longitudinal studies. This difference according to the research type suggests that we should avoid simply understanding the meta-analysis results. In addition, the subgroup analysis of published continental social network interventions showed that depression by social network intervention decreased in Asia compared to the control group, but similar results were not found in the West.

In other words, the results of the subgroup meta-analysis conducted in this study can be presented as follows: First, as suggested in the longitudinal studies, social support, social participation, and social connection/network were all effective interventions for depression among the elderly in the community. Second, interventions using social networks can be more effective in Asian countries than in Western countries. Based on this, the following interventions are needed for nurses to promote social support for the elderly in the community. It is to create a center that can monitor the daily life of the elderly, especially the elderly living alone, centered on the community, and to provide a social support system for the elderly and to create a system that can detect high-risk groups such as depression in advance. It is necessary to make efforts to expand the important role of nurses centering on existing community health centers.

### 4.3. Strengths and Limitations

The main strength of this review is that it is the most recent and comprehensive quantitative synthesis of interventions in terms of social support for depression in the elderly. In particular, this study differs from previous studies in that a review of more effective intervention methods was conducted through meta-analysis by dividing various social supports. In other words, it can be said that the main achievement of this study was to provide practical guidelines for depression interventions for the elderly in the community.

Regarding the limitations of this study, the heterogeneity was high in the studies included in the meta-analysis. However, social support and social participation consistently produce results in the same direction, and it is judged that there will be no difficulty in drawing conclusions. Second, long-term follow-up studies on depression in the elderly are needed, therefore, the current results should be interpreted carefully. Finally, another limitation is that only studies published in English or Korean were used as targets for analysis.

## 5. Conclusions

The results of this systematic review support the effects of various social support interventions in reducing depression in the elderly living in the community. Various interventions in the reviewed study were divided into social support, social participation, and social connection/network, and a meta-analysis was conducted. According to the meta-analysis, social support was effective in reducing depression among the elderly in the community; therefore, nurses should allow the elderly in the community to create a system that allows them to perceive social support resources and escape negative emotions such as depression by experiencing emotional support and exchange through social participation. However, not all social resources for depression among the elderly in the community can be provided by nurses; hence, it is necessary to speak out so that related social systems can be established.

## Figures and Tables

**Table 1:** The characteristics of the selected studies [3,4,5,6,7,8,9,10,11,12,13,14,15,16,17,18,19,20,21,22,23,24,25,26,27,28,29,30,31,32,33,34,35,36,37,38,39,40,41,42].

Author (Year)	Study Design	Country	Object Country	Sample Size (*n*)	Age (Mean, Range)	Location	Male/Female (*n*)	Depression Measurement	Social Support Measure	Social Support Explanation	Covariate
		(City)									
Mulat (2021)	cross-sectional	Ethiopia	Ethiopian	959	69.04 (SD 6.602)	Community (urban/rural)	463/478	GDS	Perceived social support: the Oslo-3 scale and individuals score	Perceived social support: social support has been described as support access to an individual through social ties to other individuals, groups, and the larger community	age, gender, occupational status, marital status, family size, living arrangement, known chronic disease, physical disability, sleep medication, a good relationship with neighbors, feeling of loneliness, ever used tobacco
Choi (2020)	cross-sectional	Korea	Korean	4751 Depressed1280 Non-depressed3471	Depressed 73.82 (SD 7.90) Non-depressed71.24 (SD7.42)	Community	Depressed 421/859 Non-depressed1512/1959	CES-D	Social participation, Emotional social support: Additional survey of the Korean Retirement and Income Study (KReIS)	The social participation (1) economic activity (2) social activity (3) volunteer work (4) donation Emotional social support: Eight items were used to measure perceived emotional social support (e.g., “I have someone to talk to when I am lonely”, “Others comfort me when I am upset”, “I have someone to trust and rely on”)	age, gender, education level, income level, marital status, living alone, chronic disease, self-rated health, limitations on activities of daily living, satisfaction with living conditions
Adams (2020)	cross-sectional	Tanzania	Tanzanian	304	60–80, >80	Community (rural)	149/155	GDS-15	the Oslo-3 Social Support Scale (OSS-3)	The scale provides a brief measure of social functioning. (1) how many people are so close to you that you can count on them if you have great personal problems? (none, 1–2, 3–4, and 5 or more), (2) how much interest and concern do people show in what you do? (none, little, uncertain, some, and a lot) (3) how easy is it to get practical help from neighbors if you should need it? (very difficult, difficult, possible, easy, and very easy)	age, gender, education, occupation, marital status, living alone, participation in social activities, participation in religious activities, consumed alcoholic drink past 12 months, ever consumed tobacco products, history of hypertension, history of stroke, history of diabetes, stressful life events past one year, history of cognitive impairment, family history of depression
Ahmad (2020)	cross-sectional	Malaysia	Malaysian	3772	over 60	community	1872/2105	Malay version of the Geriatric Depression Scale (M-GDS-14)	Duke’s Social Support Index	Duke’s Social Support Index: scores of 11–26 were considered as low social support.	locality, highest education level, sex, living arrangements
Bal (2020)	cross-sectional	China	Chinese	1810	70 (SD 7.51) (range 60–96)	community	770/1040	The Zung self-rating Depression Scale (SDS)	The framework of the World Bank’s Social Capital Assessment Tool and previous works of our research group: six dimensions of social capital	(1) social participation (2) social support (3) social connection (4) trust (5) cohesion (6) reciprocity	age, gender, BMI, residence, living status, marital status, education, smoking, drinking status
Bui (2020)	longitudinal	United states	American	2200	67.235 (SD 0.229) (range 57–85)	community	48% male 52% female	CES-D	Social support, Network structure Social network function	Network structure (1) network size (2) the number of close ties (3) frequency of contact with alters (4) density Social network function (1) network function: network function was operationalized as social support experienced from friends and family. Here were four variables in total—two each for family and friends. (2) Social support scale: scale was created by summing the responses for the four questions asking if the respondent could rely on or open up to family and friends.	depressive symptoms, age, female, white, college or higher, cohabiting
Jin (2020)	cross-sectional	China	Chinese	1779	69.22 (SD 6.98)	community	585/1194	GDS-5	Social Support Rating Scale (SSRS)	(1) objective support (e.g., what are the sources of help you receive when you are in trouble?) (2) subjective support (four items) (e.g., how many close friends do you think you can rely on for help when you need it?) (3) support utilization (three items) (e.g., do you seek help when in trouble?).	age, female, high income, years of schooling, cognitive impairment, number of chronic diseases, ADL score, IADL score, pain, physical frailty score
Kim (2020)	Prospective cohort	America	American	2261	68.5 (SD = 7.5) 57–85 (range)	community	48%/52%	CES-D	(1) Network size (Individual-level) (2) Social interaction (Individual-level)	(1) Network Size: the number of close friend and family/relatives (six-point scales, 0 = ‘none’ to 5 = ‘more than 20’) (2) Social interaction: the level of frequency of social interaction (1 = ‘less than once a year’, to 6 = ‘several times a week)	-
Lee (2020)	cross-sectional	Korea	Korean	10,082	over 65	community	4046/6036	The Korean version of the Geriatric Depression Scale-Short form (SGDS-K)	Emotional support exchange Social network Social participation	(1) Emotional support exchange: The degree to which emotional help is exchanged with cohabitation, non-habitation, parents, and spouses respectively. (2) Social network: Number of family members, friends, neighbors, and acquaintances who are close (3) Social participation: Clubs’, ‘social groups’, ‘political and social group activities’, ‘volunteer activities’, ‘religious activities’	education, equivalent household income
Reynolds (2020)	longitudinal	United states	American	1592	69.3 (SD 7.9) (range 57–85)	community	48% male 52% female	Epidemiologic Studies Depression Scale	community-layer connection interpersonal-layer connection partner-layer connection	(1) community-layer connection: “level of attendance at meetings of any organized group such as a choir, a committee or board, a support group, a sports or exercise group, a hobby group, or a professional society.” “How often they do volunteer work for religious, charitable, political, health-related, or other organizations”. (2) interpersonal-layer connection: important people in their life, including friends and confidants with whom they interact regularly and discuss important matters, as well as people with whom they live. (3) partner-layer connection: current spouse or romantic partner	depression, functional health problem, age, job status, assets, sex, education, race: black, race: white, ethnicity: Hispanic
Wu (2020)	cross-sectional	Taiwan	Taiwanese	153	71.56 (SD 8.46)	community	57/96	GDS-15	Chinese version of the Multidimensional Scale of Perceived Social Support Chinese version of the Intergenerational Relationship Scale	(1) multidimensional scale of social support: measure perceived adequacy of social support and feelings of appropriate support received from family, friends, and significant others. (2) intergenerational relationship scale: includes items on affectual solidarity, functional solidarity, and structural solidarity dimensions.	age, sex, marital status, education, religious preference, living arrangement, employment, economic status, perceived health, comorbidity, medications, sleep quality, nap habits, regular exercise, leisure activities, Barthel index, IADL, Use of social media
Gu (2019)	cross-sectional	China	Chinese	172	74.92 (SD = 6.63) 60–92 (range)	community	62/110	GDS-15	Lubben social network scale (LSNS-6)	Family social support network (three items) and friend social support network (three items): the number of relatives or friends whom older people feel close to or ask for support (0 = ‘none’, to 5 = ‘nine or higher’) (Total score range: 0–30, If score < 12: social isolation)	Sex, Age, Educational level, Economic status, Number of chronic illnesses, cognitive function
Kim (2019)	cross-sectional	South Korea	South Korean	1000	74.9 (SD = 6.4) 65–90 (range)	community	410/590	GDS-15	Lubben social network scale Revised (LSNS-R)	(1) The size of the individual’s active social network: relatives or friends seen or heard from ≥1 times/month (2) Perceived support network: relatives or friends who could be called on for help (3) Perceived confident network: relatives or friends to whom the respondent could talk about private matters (Total score range: 0–60)	Sociodemographic variables(Age, Gender, Marital Status, Education, Income, Living arrangement, Residential area) Health-related variables (Self-rated health, Chronic diseases, IADL)
Yamaguchi (2019)	Prospective cohort	Japan	Japanese	29,065	M:72.3 (SD = 5.4) F:72.4 (SD = 5.4)	community	14465/14600	GDS-15	Social capital (1) Civic participation (2) Social cohesion (3) Reciprocity	(1) Civic participation: level of residents’ participation in community organizations and activities (2) Social cohesion: cognitive aspects of interpersonal trust, reciprocity, and attachment to the community (3) Reciprocity: the community characteristics of exchanging support	Age, Family structure, Martial Status, Income, Current employment, Educational attainment, Comorbidity
Chao (2018)	cross-sectional	America (Chicago)	Chinese American	3157	72.8 (SD = 8.3) 60–105 (range)	community urban and rural	1318/1821	PHQ-9 (The patient Health Questionnaire)	(1) Positive Perceived Social Support (6 items) (2) Negative Perceived Social Support (6 items)	(1) Positive PSS: how often they opened up to support systems to talk about their worries, and how often they relied on support systems for help (2) Negative PSS: how often they believed they had been demanded and criticized by their support systems (1 = ‘hardly ever’ to 3 = ‘often’)	Social demographic variables (age, gender, years of education completed, annual personal income, marital status, the number of children, living arrangement, years in the United States, years in the community, country of origin, medical comorbidities)
Compete (2018)	cross-sectional	Mexico city	Mexican	526	age 65 and above	community center	526 (only women)	GDS-15	Perceived social support (OSS-3; Oslo scale 3 items)	Perceived social support: the quantity and satisfaction of individuals’ perceived social networks (Total range: 3–14, Higher values represent greater support)	Elder abuse, Age, Education, Household size, Lives alone, Currently employed, Comorbidities, Self-reported health status, Functional impairment(ADL, IADL)
Gayman (2018)	cross-sectional	America (Miami-Dade)	African American	248	58.11 (SD = 16.26) 18–86 (range)	community	NS	CES-D-20	Perceived social support (a modified and shortened version of the Provisions of Social Relations scale) (1) Family support (16 items) (2) Friend support (8 items)	Perceived Social support (1) Family support (2) Friend support (higher values represent greater support)	Socioeconomic Status (Household income), Social stressors, Daily discrimination, Mastery, Self-esteem, Marital Status
Hu (2018)	Prospective cohort	China	Chinese	6772	age 60 and above	rural and urban	3390/3382	CES-D	Social support (1) Family support (2) Community support (3) Public support	(1) Family support: the older adults live with a spouse, the numbers of children and grandchildren, and the frequency of contacting with children (2) Community support: facility (whether the senior center presented), organization (whether the community had elderly association), activity (whether the community organized activities frequently) (3) Public support: social security and welfare for older adults	Individual demographics (gender, age, educational level, physical health status), The domain of family attributes (annual household expenditure per capita), Residential areas
Kim (2017)	cross-sectional	America	Japanese American	207	86.74 (SD = 6.48) 68–103 (range)	community or institutional	50/157	GDS-15	Social support (MOSS-E; The Measurement Of Social Support in the Elderly scale)	Instrumental support (assisting with physical needs such as cooking and cleaning) & Emotional support (assisting emotions and mental health) & Providing support	Demographic variables (Age, Gender, Martial status, Education, Income), Cognitive function(MMSE)
Park (2017)	cross-sectional	America	Korean American	209	69.59 (SD = 7.51)	community	75/134	CES-D-9 (short form)	Social integration variables (1) Social network (LSNS-6; Lubben social network scale) (2) Community social cohesion (5 items)	(1) Social network: same as Gu (2019) (2) Community social cohesion: the degrees of social cohesion and trust (Total range: 5–20, Higher values represent higher levels of community social cohesion)	Demographic variables (Age, Gender, Education, Perceived income, Length of stay in the USA), Health variables (Chronic conditions of 9 diseases, Functional disability-ADL, IADL), Living alone
Ang (2016)	Prospective cohort	Singapore	Chinese, Malay, Indian	2766	age 60 and above	community	1290/1476	CES-D	Received social support	Money, Housework help, Material goods (Food, Clothes or other), Mobility help (Help to go to the doctors, marketing, shopping, go out to visit friends, using public transportation), Emotional support or advice	Socio-demographics (Race, living arrangement, employment status, housing type), Functional limitation (ADL, IADL), Chronic illnesses, Difficulty with vision, Difficulty with hearing
Aung (2016)	cross-sectional	Thailand	Thai	435	83.8 ± 3.5	community urban and rural	196/239	GDS-30	Social Network Index (SNI)	the number of social roles in which the respondent has regular contact, at least once every 2 weeks, with at least one person: (12) spouse, parents, their children and children-in-law, close relatives, close friends, religious members (such as church or temple), classmates, teachers and students in adult education, coworkers or colleagues, neighbors, volunteer networks, and others organizations (Score: 1–3 (limited), 4–5 (medium), 6 and over (diverse) social network)	Demographics (age, sex, and educational attainment), Health status (dependency, self-impression of health), Cognitive decline (short-term and long-term memory loss)
Chen (2016)	cross-sectional	Hong Kong	Hong Kong	400	80.2 (SD = 7.5)	community facilities	174/226	GDS-15	Neighborhood support network (1) Family living together (2) Family and relatives (3) Friends (4) Organizations	The persons who they relied on for help in buying groceries and daily necessities, and escorting to medical appointments, without setting a limit on the number of people they named. Each person named was classified into 4 ->1), 2), 3), 4)	Age, Gender, ADL, Recent fall history, Marital status, Monthly income, Education level, Perceived proximity
Li (2016)	cross-sectional	China	Chinese	5103	68.65 (SD = 7.45) 60–101 (range)	community urban and rural	2552/2551	CES-D	Social support and participation (1) partnered status (2) children nearby (3) social participation (4) elderly activity center in community	(1) Partnered status: married/cohabiting or not (2) Children nearby lived with children or had children living in the same community (3) Social participation: Engaged in any of following 7 activities (spent time with friends; played cards, chess, or ma-jong with others; provided help to non-core-siding family members, friends or neighbors; visited a park or a social center to dance/exercise; participated in activities organized by community organizations; participated in volunteer work; and attended a class or training workshop) (4) Elder activity center: opportunities for social interaction and participation.	Age, Gender, Are (Rural-urban), Socioeconomic status (Education, Pension benefit, Household asset, Community infrastructure), Healthcare access (Distance to healthcare facility, health insurance, No physician visit when ill, No hospitalization when needed, Self-discharge from hospital), Health Status (Chronic conditions, ADL, IADL)
Tsuboi (2016)	cross-sectional	Japan	Japanese	24,632	65–100 (range)	community	11,869/12,763	GDS-15 (Japanese ver.)	Social support (the 2-Way Social Support Scale) (1) Receiving emotional support (RES) (2) Giving emotional support (GES) (3) Receiving instrumental support (RIS) (4) Giving instrumental support (GIS)	(1) RES: a person who hears a respondent’s complaints or worries (2) GES: a person who shares his/her complaints or worries with respondent (3) RIS: a person who would nurse or take care of the respondent (4) GIS: a person whom the respondent would nurse or take care of were he/she ill in bed for several days.	ADL, Socioeconomic status (years of schooling, annual income), living alone
Vanoh (2016)	cross-sectional	Malaysia	Malaysian	2264	With depressive: 69.8 (SD = 6.4) without:68.9 (SD = 6.2)	community	1083/1181	GDS-15	Medical Outcome study Social Support (MOSS)	Assessing social support (not specific)	Sociodemographic, Calorie restriction, Fitness, Health status, Functional status, Cognitive status, Lifestyle activities
Yoo (2016)	cross-sectional	South Korea	South Korean	648	75.4 (SD = 5.9)	community (Homes, Small community halls, senior welfare centers)	195/453	SGDS-K (KoreanversionofGDS-15)	Social support (PSSS; The Perceived Social Support Scale)	PSSS (informational, tangible, emotional support and self-esteem) (Total range: 20–80, Higher values represent greater support)	Background characteristics (Age, Gender, Education, Financial activities, Current health status, Coresident family members), Physical variables (Number of chronic diseases, Functional independence; K-MBI), Psychological variables (Number of stressful life events (in the past year), Life satisfaction)
Jinhui Li (2015)	cross-sectional	Singapore	Singaporean	162	72.19 (SD = 6.23)	community urban (senior activity centers)	39/123	GDS-15	Social support (DSSI-10; Duke social support index)	DSSI-10: Social satisfaction and social interaction (Total range: 10–30, Higher values represent greater support)	Demographic data (Age, Gender, Education, Living arrangement), Perceived income adequacy, Perceived life quality, Psychological resilience (RAS), Loneliness (ULS-8)
Ng (2014)	cross-sectional	Singapore	Malay, Chinese, Indian, Others	2447	age 60 and above	community	1048/1399	GDS-15	Social support (1) Living arrangement (2) Frequency of leisure time spent (3) Social isolation	(1) Living arrangement (2) Frequency of leisure time spent: the frequency to contact with family members (3) Social isolation: the perception of being socially isolated	Chronic Diseases, Functional Status, Pain, Cognition
Wee (2014)	cross-sectional	Singapore	Singaporean	559	age 60 and above	community	250/309	GDS-15	Social network (LSNS-6; Lubben Social Network Scale)	Social network: same as Gu (2019)	Demographic factors (Marital Status), Clinical factors (Falls, visual impairment, musculoskeletal conditions, diabetes mellitus)
Chen (2012)	Prospective cohort	China	Chinese	1275	age 60 and above	community urban	490/785	SCID interview (Structured Clinical Interview for DSM-IV), PHQ-9	Social support from family (1) Family social support (LSNS; Lubben Social Network Scale) (2) Living status	(1) Family social support: 6 items, total score 0–30 (2) Living status: living alone, living with somebody (including family members and relations)	Sociodemographic (Gender, Education level), Health status(medical burden-CIRS, daily life function-IADL)
Gong (2012)	cross-sectional	China	Chinese	1317	68.67 (SD = 6.54)	community rural	655/662	BDI-II (Back Depression Inventory-II)	Support from family members	Support from family members: Asked respondents to rate support from five types of family member (spouse, parents, sons and/or daughters, siblings, and other relatives) (3 levels: Bad, Fair, Good)	Demographic(Age, gender, years of schooling), Self-perceived physical health, Family characteristics(Living with spouse, Living with descendant, Self-reported family economic status, Family-related negative life events)
Kim (2012)	cross-sectional	South Korea	South Korean	263	age 65 and above M:71.0 ± 5.8 F:74.4 ± 6.6	community	103/160	SGDS (Short form of Geriatric Depression scale-Korean ver.)	(1) Family & Friend support (12 items) (2) Social support (20 items)	(1) Family & Friend support (Higher values represent greater support of family or friend) (2) Social support: informative, material, emotional, self-esteem support	Disease stress, Economic stress, Perceived health status, Education level, Age, Hypertension
Wang (2012)	cross-sectional	China	Chinese	209	Depressed: 64.5 ± 2.86 Not-depressed:63.8 ± 2.84	community urban	98/111	GDS	Multidimensional Scale of Perceived Social Support (MSPSS) (1) Family support (2) Friend support (3) Other support	Multidimensional Scale of Perceived Social Support (MSPSS): Social support from friends, family and significant others (Higher scores indicate lower perceived support)	Family functioning (PS-Problem solving, CM-communication, RL-Roles, AR-Affective responsiveness, AI-Affective involvement, BC-Behavioral control, GF-General functioning), Marital status
Chan (2011)	cross-sectional	Macau	Chinese	839	71.4 (SD = 7.7) Median:70 (60–98)	community	NA	GDS-15	Lubben Social Network Scale (SNS)	Lubben Social Network Scale (SNS) (1) Family network (2) Networks of friend (3) helping others (4) confidence in relationships (5) living arrangements	Demographic factors (Age, Education, Ethics group, Marital status, Live status, Ability to meet living costs, Monthly income, Need spectacles, Need a hearing aid), Daily activity factors ((MBI, Ability to do the following tasks), Health needs/behavior factors (Chronic illness, Symptoms in the previous three months, Perceived health)
Chao (2011)	Prospective cohort	Taiwan	Taiwanese	1743 (2003yr)	87.1 (SD = 4.6) (2003yr)	community	926/817	CES-D	Social support (1) Social network size (2) Social network composition (3) Frequency of social contact (4) Proximity of support (5) Types of support received: Receiving instrumental support etc. (6) Helping others: Providing financial support etc. (7) Satisfaction with social support	(1) Social network size: asked to identify their marital status and count the number of family members (2) Social network composition (3) Frequency of social contact: mean frequency of meeting with children who were not living with respondents. (4) Proximity of support: whether respondents live with a married son (5) Types of support received: Receiving instrumental/emotional/financial support (6) Helping others: Providing financial/Short-term instrumental/Long-term instrumental support (7) Satisfaction with social support: how satisfied they were with the emotional support provided by families or friends	Demographic (Age, Gender, Education, Ethnicity), Physical health status (IADL)
Chan (2010)	cross-sectional	Singapore	Singaporean, Chinese, Malays, Indians, others	4489	69.3 ± 7.2 60–97 (range)	community	2078/2411	11-item CES-D	Living arrangement Modified Lubben’s revised social network scale (LSLS-12)	Living arrangement LSLS-12: Social networks with friends and with relatives outside the household (1) assessing the size of network (2) frequency of contact (3) closeness and perceived support from friends and relatives outside of the household	Living arrangements, Ethnic group, Education, Presence of ADL limitations, Presence of IADL limitation, Housing type, Social activities
Suttajit (2010)	cross-sectional	Thailand	Thai	1104	60–79, over80	community rural	495/609	EURO-D	The scale of Six Social Support deficits	(1) Living alone without a child or other relative (2) Seeing a child or other relative less often than once per week (3) Lack of reciprocity with neighbors, through asking about amount to which neighbors depend on each other in their village (4) Lack of reciprocity between children and extended family members, through asking about amount to which children and relatives care about each other (5) Difficulty in relationship with one or more relatives, through asking about severe problems in relationships between the participant and any of their children or relatives in the last year lasting more than a few weeks (6) Dissatisfaction with support from children	Age, Gender, Marital status, Education, Socioeconomic status, Work status
Chan (2009)	cross-sectional	Macau	Chinese, Asian, European, American	1042	71.4 ± 7.4 median 71.0 60–98 (range)	community	NA	GDS-15	Lubben Social Network Scale (LSNS)	Lubben Social Network Scale (SNS) (1) Family network (2) Networks of friend (3) helping others (4) confidence in relationships (5) living arrangements	Demographic factors (Age, Education, Ethics group, Marital status, Live status, Ability to meet living costs, Monthly income, Need spectacles, Need a hearing aid), Daily activity factors ((MBI, Ability to do the following tasks), Health needs/behavior factors (Chronic illness, Symptoms in the previous three months, Perceived health, Required to pay for the consultation fee)
Mechakra-Tahiri (2009)	cross-sectional	Canada	Canadian	2670	65–84, over 85 (range)	Community	1073/1596	ESA Diagnostic Questionnaire and based on the DSM-IV(ESA-Q)	Social relationship: Structural relationship (Informal network, Formal network), Functional relationship (social support, presence of conflict)	Structural relationship (1) Informal network: marital status, presence or absence of children, siblings and friends, (2) formal network: examining respondents’ participation in three community activities(visiting a social center, attending a place of worship or volunteering in community associations Functional relationship (3) Social support: Presence of confidents, instrumental support, emotional support (4) the presence of conflict: children, spouse	Age, Area of residence, Chronic condition, Self-rated health
Shin (2008)	cross-sectional	Korea	Korean	787 NSS (Normal social support):592 PSS (Poor social support):195	NSS:75.61 ± 08.44, PSS:74.89 ± 08.32	community	NSS: 52.7% (female) PSS:52.8% (Female)	DSM-IV criteria, Korean version of the Geriatric Depression Scale(GDS-K) Korean version of the Hamilton Depression Rating Scale(HAM-D)	Medical Outcome Study Social Support Survey (MOS-SSS)	(1) Functional social support (2) Including 4 subcategories of emotional/information support (3) Tangible support (4) Positive social interaction and affectionate support	Age, Gender, Education
Leung (2007)	cross-sectional	Taiwan	Taiwanese	507	72.26 (SD = 4.70) 65–92 (range)	community industrial city/rural	321/186	Chinese version of Symptom Checklist 90-R(SCL-90-R)	Social Support Rating Scale(SSRS) Chinese modification of the Family Emotional Involvement and Criticism Scale (FEICS)	SSRS: Perceived instrumental and emotional support FEICS: Family functioning	Age, Gender, Location, ADL, Cognitive function, Chronic disease, Intimacy, Criticism
Chen (2005)	cross-sectional	China	Chinese	1600	60–80, over 80	rural	754/846	Geriatric Mental State(GMS), Automated Geriatric Examination for Computer Assisted Taxonomy(AGECAT)	Social support (1) Quality (2) Quantity (3) Community participation	(1) Quality(good relationships with neighbors, parents, or others; ease in acquiring friends; and available help when needed) (2) Quantity(marital status, residence with family members, frequency of visiting children or other relatives, and contact with neighbors or friends in the village) (3) Community participation(having any religious belief and taking part in activities and participating in community activities for seniors)	(1) Basic Characteristics: Gender (2) Socioeconomic Status Indicators: Currently family income, Consumption of meat including fresh and salted meat and fish during the past year, watching television (3) Health Status: Self-assessed physical health status, Hypertension (4) Adverse Life Events Occurring in the Past 2 Years: Anything else severely upsetting, Horrifying experience, including accident, fire, physical attack, etc.
Chi (2005)	cross-sectional	Hong Kong	Chinese	917	over 60	community households	445/472	GDS-15	Lubben Social Network Scale (LSNS)	LSNS: Social support from family members and friends (1) family network (2) friend networks (3) helping others (4) confidant relationships (5) living arrangements	(1) Sociodemographic (Gender-male, Age 75+, Residence in Hong Kong years < 20 years, Married, Living alone, Employed, Attained high school education, Having religious belief) (2) Health status (Poor self-rated health status, Subjective long-term pain, Vision problem, Severity of ADL impairment) (3) Financial situation (Self-rated financial strain)
Koizumi (2005)	Prospective cohort	Japan	Japanese	753	over 70	community urban	NA	GDS	Social support questionnaire	Social support: (1) To consult in trouble (2) To consult in bad physical condition (3) To help with your daily housework (4) To take to a hospital (5) To take care of you	sex, age, GDS score in the 2002 CGA, presence or absence of spouse, number of household members, number of past physical diseases, age at finishing school education, MMSE score, physical function, pain, self-rated health
Lee (2005)	cross-sectional	Korea and Japan	Korean and Japanese	K:1298/J:1495	over 65	community	K: 60.3% (female) J: 60.8% (female)	GDS-15	Social support index: Comprised of both receiving and giving social support	Comprised of both receiving and giving social support	Age, gender, Education, Poor self-rated health, Functional capacity, Cognitive impairment, Smoking, Sleep, BMI, Hospitalization, lifetime occupation, Chronic condition
Tsai (2005)	cross-sectional	Taiwan	Taiwanese	1200	With:74.6 (SD = 5.6) without:74.3 (SD = 5.4)	community	with:164/166 without:506/364	GDS-15	Social support scale (1) Social support network (2) Quantities of social support (3) Satisfaction of social support	Social support scale: social support among elders living alone (1) Social support network: number of relatives or friends who would likely contact the elder and by the quantity of contacts during the previous week. (2) The quantities of social support: asking participants to rate each social behavior offered by different providers. (3) Satisfaction with social support: the level of satisfaction with quantities of support and support resources in general.	gender, educational level, marital status, number of diseases, satisfaction with living situation, perceived health status, perceived income adequacy, cognitive status, functional status, disease
Adams (2004)	cross-sectional	America	American	234	81.35 ± 7.0 60–98(range)	Independent living section of congregate retirement housing (Residentsaregenerallyretiredandwithoutadultchildrenorgrandchildrenlivinginthesamehousehold)	56/159 (not respond:19)	GDS	Lubben Social Network Scale(LSNS) Number visitors/week Visitor type	Lubben social Network Scale (1) Family or relative networks (2) Friend networks (3) Confidant relationships (4) Helping relationships (5) Living arrangements Number visitors/week Visitor type: neighbor, visitor: Adult child, Visitor: Friend	Age, Gender, Marital status, Facility, Number of chronic health conditions, Grieving, Number activities/week, Church attendance/month, UCLA Loneliness Scale
Chi (2001)	cross-sectional	Hong Kong	Chinese	1106	72.55 (SD = 7.33) 60–95 (range)	community	488/618	CES-D	social support	Social support (1) Social network size (2) Network composition (3) Social contact frequency (4) Satisfaction of social support (5) Instrumental/emotional support (6) Helping others	Demographic (Age, Gender, Years of education), Functional impairment (ADL, IADL, Physical performance)
Hays (1998)	cross-sectional	America	American	4162	72.92 (SD = 6.29) 64–100(range)	Community Household	NA	CES-D	Perceived social support	(1) availability of at least one trusted confidant and satisfaction with the amount of social interaction (2) Social interaction frequency: numbers of friends and relatives seen or telephoned in the past month and memberships in clubs or organizations (3) Instrumental support given to and received from family/friends: Items included such domains as providing meals or transportation, loaning money, and giving advice or gifts. (4) Social network size: concerning numbers of friends/relatives respondent’s social network	Age, Gender, Race, Years of education, Family income, Cognitive impairment, Chronic health problems, Functional disability, Negative life events
Antonucci (1997)	cross-sectional	France	French	3777	75.21 (SD = 6.92)	community urban	1576/2201	CES-D	Social relation: version of portions of the Social networks in Adult life Questionnaire	(1) Size of their network (2) the Composition of their network (3) how many people in their network do not understand them (4) Satisfied with the quality of their relationships with their network	Age, Gender, Functional impairment
Henderson (1997)	Prospective cohort	Australia	Australian	1045	80.1 (SD = 4.9) 73–102 (range)	community Wave1:communityorinstitution	NA	Canberra Interview for the Elderly (CIE) (ICD-10 andDSM-III-RorDSM-IV)	Social support	(1) close friends, reflects whether subjects had people to whom they felt close and from whom they could ask help and support. (2) social visit, reflects the amount of visiting to and from family and friends, neighbors and clubs	(1) Depression score (2) Sociodemographic variables (Age, Gender) (3) Level of education, Psychological health variables, Physical health variables (ADL, Number current symptoms, Number medical conditions, Blood Pressure, Global health rating, Sensory impairment) (4) Services used (community sample only)

## Data Availability

The data presented in this study are available on request from the corresponding author.

## Supplementary Materials

The following supporting information can be downloaded at: https://www.mdpi.com/article/10.3390/healthcare10091598/s1, Table S1: Characteristics of the subjects presented in each study; Table S2: Various depression intervention methods.
