# Supplementary material for: Effectiveness of Social Support for Community-Dwelling Elderly with Depression: A Systematic Review and Meta-Analysis
Source: Healthcare (Basel). 2022 Aug 23;10(9):1598. doi: 10.3390/healthcare10091598 (PMC9498411; doi:10.3390/healthcare10091598)
Supplement: Supplementary file 1 [file healthcare-10-01598-s001.zip › healthcare-1868884-supplementary.pdf]

Table S1. characteristics of the subjects presented in each study.

	Author	Year	Variable		Coefficient(B)	Standard Error(SE)	Standardized Coefficient(β)	t	OR	95%CI	p	Parameter estimate
Age	Mulat	2021	Age	>=75years					7.95	4.98-12.68	<0.001	
				70-74years					5.52	3.52-8.66	<0.001	
				65-69years					2.39	1.54-3.70	<0.001	
				60-64years					1			
	Bui	2020	Age		0.029	0.015			1.03	1.00-1.06		
	Jin	2020	Age				-0.178				<0.001	
	Kim	2020	Age(in decades)		0.001	0.008			1.00	0.99-1.02		
	Raynolds	2020	Age				0.3				<0.001	
	Wu	2020	Age		0.02	0.05		0.32	1.02	0.92-1.13	0.752	
	Kim	2019	Age		0.02	0.019	0.02		1.02	0.98-1.06		
	Chao	2018	Age	Mild					1.01	(0.99,1.02)		
				Moderate severe					1.03	(1.01,1.05)	<0.01	
	Compte	2018	Age						1.02	(0.99,1.06)		
	Hu	2018	Age 70-79 (Ref 60-69)	Urban					0.698	(0.519,0.938)	<0.05	
				Rural					1.004	(0.868,1.161)		
				All					0.885	(0.779,1.005)	<0.10	
			Age 80 and over (Ref 60-69)	Urban					0.679	(0.401,1.153)		
				Rural					0.828	(0.638,1.075)		
				All					0.731	(0.581,0.920)	<0.01	
	Kim	2017	Age				-0.26	-3.86			<0.01	
	Park	2017	Age				0.19				<0.05	

	**Aung**	**2016**	Age		-0.04				0.96	(-0.09, 0.00)	0.07	
	**Chen**	**2016**	Age		0.01	0.02	0.01		1.01	0.97-1.05		
	**Li**	**2016**	Age		-0.025		0.012		0.98	0	<0.05	
	**Yoo**	**2016**	Age				0.07				0.21	
	**Jinhui Li**	**2015**	Age				-0.01					
	**Gong**	**2012**	Age						0.99	(0.96,1.01)		
	**Kim**	**2012**	Age(≥75)			0.57	-1.41				0.015	
	**Chao**	**2011**	Age		-0.004	-0.001			0.996	0.994-0.998	<0.001	
	**Chan**	**2011**	Age(in years)	Men		(0.01)	-0.004					
				Women		(0.01)	-0.02					
	**Tahiri**	**2009**	70-74 versus 65-69	Metropolitan					0.63	(0.38,1.04)		
				Urban					0.52	(0.26,1.06)		
				Rural					0.48	(0.30,0.76)		
				Total population					0.53	(0.40,0.73)		
			75-70 versus 65-69	Metropolitan					0.68	(0.40,1.15)		
				Urban					0.40	(0.18,0.89)		
				Rural					0.44	(0.27,0.71)		
				Total population					0.51	(0.37,0.71)		
			80-84 versus 65-69	Metropolitan					0.30	(0.15,0.62)		
				Urban					0.70	(0.32,1.51)		
				Rural					0.34	(0.20,0.58)		
				Total population					0.39	(0.27,0.56)		
			85+ versus 65-69	Metropolitan					0.43	(0.19,1.00)		
				Urban					0.28	(0.05,1.49)		
				Rural					0.68	(0.29,1.60)		

				Total population					0.47	(0.27,0.81)		
	**Leung**	**2007**	Age			0.044						0.003
	**Chi**	**2005**	Older age (75+)						0.690	(0.395,1.203)	0.191	
	**Lee**	**2005**	Age 70-74(vs 65-69)	Anyang					1.16	(0.66,2.04)		
				Yoita					0.71	(0.44,1.14)		
				Total					0.94	(0.66,1.33)		
			75-79	Anyang					1.09	(0.56,2.13)		
				Yoita					0.72	(0.43,1.21)		
				Total					0.85	(0.58,1.26)		
			≥80	Anyang					0.61	(0.29,1.31)		
				Yoita					0.89	(0.50,1.58)		
				Total					0.78	(0.51,1.21)		
	**Adams**	**2004**	Age		0.119	0.045			1.13	1.03-1.23	0.008	
	**Chi**	**2001**	Age		-0.56	0.04	-0.041		0.57	0.53-0.62		
	**Heys**	**1998**	Age		0.001				1.00			
	**Antonucci**	**1997**	Age			0.02					<0.02	-0.05
	**Henderson**	**1997**	Age				-0.024				0.528	
**Gender**	**Mulat**	**2021**	Sex	Female					1.6	1.15-2.23	0.005	
				Male					1			
	**Bui**	**2020**	Gender	Female	0.415	0.211			1.51	1.00-2.29		
	**Jin**	**2020**	Gender	Female	0.032				1.03		0.171	
	**Kim**	**2020**	Gender	Female	0.006	0.012			1.01	0.98-1.03		
	**Reynolds**	**2020**	Sex		0.1				1.11		<0.001	
	**Wu**	**2020**	Sex		-1.06	0.62		1.71	0.35	0.10-1.17	0.9	
	**Kim**	**2019**	Gender	Male	0.17	0.294	0.02		1.19	0.67-2.11		
	**Chao**	**2018**	Gender	Mild					1.44	(1.12,1.86)	<0.01	

				Moderate severe					1.81	(1.32,2.48)	<0.001	
	**Hu**	**2018**	Male	Urban					0.532	(0.404,0.701)	<0.001	
				Rural					0.588	(0.515,0.671)	<0.001	
				All					0.594	(0.528,0.668)	<0.001	
	**Kim**	**2017**	Gender	Male			-0.02	-0.33				
	**Park**	**2017**	Gender	Female			0.12					
	**Aung**	**2016**	Sex				0.02			(-0.31,0.36)	0.90	
	**Chen**	**2016**	Gender	Male	0.48	0.36	0.07		1.62	0.80-3.27		
	**Li**	**2016**	Gender	Male	-1.119	0.178			0.33	0.23-0.46	<0.001	
	**Yoo**	**2016**	Gender	Female			-0.05				0.39	
	**Jinhui Li**	**2015**	Gender				-0.05					
	**Chen**	**2012**	Gender (Ref. Male)	Female	0.693	0.393			2.01	(0.93,4.32)	0.043	
	**Gong**	**2012**	Gender (Ref. male)	Female					1.18	(0.85,1.65)		
	**Chao**	**2011**	Gender		0.085	(0.014)			1.09	1.06-1.12	<0.001	
	**Tarihi**	**2009**	Gender(Women vs. Men)	Metropolitan					2.47	(1.53,3.97)		
				Urban					1.25	(0.70,2.26)		
				Rural					3.22	(2.14,4.86)		
				Total population					2.5	(1.90,3.31)		
	**Leung**	**2007**	Gender			0.435					<0.001	-1.761
	**Chen**	**2005**	Gender	Male					1.00			
				Female					2.19	(1.31,3.67)	<0.01	
	**Chi**	**2005**	Gender	Male					0.645	(0.369,1.125)	0.122	

	**Lee**	**2005**	Women	Anyang					0.84	(0.43,1.63)		
				Yoita					1.14	(0.78,1.66)		
				Total					1.08	(0.78,1.50)		
	**Adams**	**2004**	Gender		1.106	0.745			3.02	0.70-13.02	0.140	
	**Chi**	**2001**	Gender		1.171	0.64	0.058		3.23	0.92-11.31		
	**Hays**	**1998**	Gender	Female	0.27				1.31		<0.001	
	**Antonucci**	**1997**	Gender	Male		0.27					<0.001	3.85
	**Henderson**	**1997**	Sex				0.034				0.370	
**Ethnicity/Race**	**Ahmad**	**2020**	Ethnicity	Malay					R			
				Malaysian Chinese					0.87	0.50-1.51	0.624	
				Malaysian Indian					0.83	0.25-2.72	0.76	
	**Bui**	**2020**	Race	White	0.148	0.228			1.16	0.74-1.81		
	**Kim**	**2020**	Race	White	0.013	0.016			1.01	0.98-1.05		
	**Reynolds**	**2020**	Race	Black	-0.07				0.93			
				White	-0.06				0.94			
			Ethnicity	Hispanic	-0.1				0.90		<0.05	
	**Chao**	**2011**	Ethnicity		-0.017	(0.018)			0.98	0.95-1.02		
	**Chan**	**2010**	Malay	Men		(0.2)	-1.03				<0.05	
				Women		(0.2)	1.1				<0.05	
			Indians	Men		(0.2)	0.1					
				Women		(0.3)	0.7				<0.05	
	**Hays**	**1998**	Race	African-American	-0.07				0.93			
**Marital status**	**Mulat**	**2021**	Marital status	Single					0.82	0.16-4.19	0.82	
				Divorced					2.53	1.59-4.03	<0.001	
				Widowed					2.65	1.61-4.34	<0.001	

				Married					1			
	**Ahmad**	**2020**	Marital status	Married/have partner					R			
				Widowed/no current partner					1.5	1.18-1.90	0.001	
	**Kim**	**2020**	Married		-0.035	0.013			0.97	0.94-0.99	<0.01	
	**Wu**	**2020**	Marital status		-2.04	0.79		2.59	0.13	4.19-4.45	0.011	
	**Kim**	**2019**	Married		0.11	0.345	0.01		1.12	0.57-2.20		
	**Chao**	**2018**	Marital status	Mild					0.88	(0.79,0.98)	<0.05	
				Moderate severe					0.82	(0.72,0.93)	<0.01	
	**Gayman**	**2018**	Marital Status	Separated		2.08	0.12				<0.05	
	**Kim**	**2017**	Married				0.04	0.77				
	**Chen**	**2016**	Married		0.08	0.40	0.01		1.08	0.49-2.37		
	**Wee**	**2014**	Married						0.44	(0.27,0.74)	0.002	
	**Wang**	**2012**	Marital status		-1.914				0.15	(-0.181,3.069)	0.036	
	**Chi**	**2005**	Married						0.930	(0.530,1.632)	0.800	
	**Adams**	**2004**	Marital status		-0.960	0.842			0.38	0.07-1.99	0.256	
	**Chi**	**2001**	Marital status		-1.343	0.65	-0.067		0.26	0.07-0.93	<0.05	
**Living arrangement**	**Mulat**	**2021**	Living arrangement	Children					0.85	0.56-1.30	0.469	
				Alone					1.3	0.32-5.29	0.712	
				Spouse					1			
	**Bui**	**2020**	Cohabiting		-0.617	0.242			0.54	0.34-0.87	<0.05	
	**Wu**	**2020**	Living arrangement		1.85	1.4		1.32	6.36	0.41-98.89	0.19	
	**Kim**	**2019**	Living with family		-0.83	0.375	-0.10		0.44	0.21-0.91	<0.05	
	**Chao**	**2018**	Living arrangement	Mild					1.01	(0.95,1.08)		

				Moderate severe					1.00	(0.92,1.08)		
	**Compte**	**2018**	Lives alone						1.20	(0.71,2.03)		
	**Hu**	**2018**	Living with a spouse	Urban					0.678	(0.491,0.937)	<0.05	
				Rural					0.814	(0.704,0.940)	<0.01	
				All					0.777	(0.682,0.885)	<0.001	
	**Park**	**2017**	Lives alone				0.14				<0.05	
	**Yoo**	**2016**	Coresident family members	Living with only partner			-0.11				0.06	
				Living with partner/child			-0.12				0.03	
				Living with relatives/others			0.06				0.25	
	**Jinhui Li**	**2015**	Living arrangement				-0.05					
	**Ng**	**2014**	Living arrangement (Ref. Spouse with child(ren)/grandchild(ren))	Spouse only					1.05	(0.57,1.93)	0.879	
				Child(ren)/grandchild(ren) only					0.74	(0.31,1.77)	0.494	
				Alone or with domestic helper					2.73	(1.31,5.69)	0.007	
				Others (relatives, friends, tenant)					1.82	(0.78,4.24)	0.166	
	**Chen**	**2012**	Living status (Ref. with someone)	Alone or with domestic helper	0.551	0.373			0.57	(0.68,1.68)	0.035	
	**Gong**	**2012**	Living with spouse (Ref. Yes)	No					0.80	(0.56,1.14)		

			Living with descendant (Ref. Yes)	No					1.02	(0.73,1.42)		
	**Chan**	**2010**	Living alone	Men		(0.3)	1.2				<0.05	
				Women		(0.2)	1.7				<0.05	
			Living with at least one child, no spouse	Men		(0.2)	0.8				<0.05	
				Women		(0.2)	0.4				<0.05	
			Living with a spouse, no child	Men		(0.1)	-0.3				<0.05	
				Women		(0.2)	-0.03					
			Living only with others	Men		(0.3)	0.3					
				Women		(0.3)	0.4					
	**Suttajit**	**2010**	Living alone without a child or other relative						0.7	(0.4,1.1)		
	**Shin**	**2008**	Cohabitation						0.73	(0.36,1.51)	0.40	
	**Chen**	**2005**	Living with spouse, children, or grandchildren						1.00			
			Living with parents						4.31	(1.27,14.70)	<0.05	
			Living with no one						1.98	(1.15,3.42)	<0.05	
	**Chi**	**2005**	Living alone						1.072	(0.522,2.202)	0.850	
**Economic**	**Employment**											
	**Mulat**	**2021**	Occupational status	Retired					0.7	0.38-1.28	0.247	
				Merchant					0.41	0.13-1.30	0.131	
				Others(/gov/t,NGO/daily labor)					0.62	0.37-1.02	0.064	
				Farmer					1			

	**Choi**	**2021**	Economic activity -Yes	All					0.97	0.82-1.14		
				Female					0.87	0.70-1.08		
				Male					1.14	0.87-1.49		
			Economic activity -No	All					1			
				Female					1			
				Male					1			
	**Kim**	**2020**	Employed		-0.028	0.012			0.97	0.95-0.996	<0.05	
	**Reynolds**	**2020**	W1 Job status		-0.04				0.96		<0.05	
	**Wu**	**2020**	Employment		-0.15	0.74		0.2	0.86	0.20-3.67	0.844	
	**Compte**	**2018**	Employed						0.73	(0.34,1.59)		
	**Yoo**	**2016**	Financial activities				-0.15				<0.01	
	**Chi**	**2005**	Employed						1.191	(0.478,2.969)	0.707	
	**Income**											
	**Ahmad**	**2020**	Individual monthly income(MYR)	<1000					3.03	1.73-5.32	<0.001	
				1000-1999					2.58	1.41-4.70	0.002	
				>=2000					R			
	**Jin**	**2020**	High income		-0.007				0.99		0.776	
	**Lee**	**2020**	Equivalent household income: Highest 25%	Men					1			
				Women					1			
			Equivalent household income: 2nd 25%	Men					1.69	1.18-2.42		
				Women					1.36	1.06-1.75		
			Equivalent household income: 3rd 25%	Men					2.78	1.94-3.98		
				Women					1.95	1.50-2.52		

			Equivalent household income: Lowest 25%	Men					3.9	2.72-5.59		
				Women					2.96	2.29-3.81		
	**Reynolds**	**2020**	Assets		-0.1				0.90		<0.001	
	**Kim**	**2019**	Household income(Moderate)		-2.35	0.538	-0.30		0.10	0.03-0.27	<0.001	
			Household income(High)		-2.61	0.537	-0.33		0.07	0.03-0.21	<0.001	
	**Chao**	**2018**	Income	Mild					0.94	(0.83,1.05)		
				Moderate severe					0.71	(0.58,0.87)	<0.001	
	**Gayman**	**2018**	Neighborhood income			0.00	-0.09					
	**Kim**	**2017**	Income				-0.17	-2.93			<0.05	
	**Park**	**2017**	Perceived income				-0.04					
	**Chen**	**2016**	Monthly income (Ref. welfare)	HK $0-$1999	-0.65	0.63	-0.05		0.52	0.15-1.79		
				HK $2000-$3999	-0.80	0.50	-0.08		0.45	0.17-1.20		
				HK $4000-$5999	-1.02	0.46	-0.12		0.36	0.15-0.89	<0.05	
				HK $6000-$7999	-1.66	0.51	-0.16		0.19	0.07-0.52	<0.01	
				HK $8000 or above	-1.94	0.52	-0.20		0.14	0.05-0.40	<0.001	
	**Li**	**2016**	Household asset	Second	-0.870	0.231			0.42	0.27-0.66	<0.001	
				Third	-1.525	0.247			0.22	0.13-0.35	<0.001	
				Richest	-2.240	0.292			0.11	0.06-0.19	<0.001	
	**Jinhui Li**	**2015**	Perceived income adequate				-0.13				0.067	
	**Tarihi**	**2009**	Income: <15000 versus 15000+	Metropolitan					1.23	(0.73,2.07)		
				Urban					0.90	(0.41,1.97)		
				Rural					0.87	(0.56,1.34)		

				Total population					0.96	(0.70,1.29)		
	**Shin**	**2008**	Low income						1.73	(0.95,3.16)	0.07	
	**Chen**	**2005**	current family income(estimated mean, US $)	Very satisfactory or satisfactory($402)					1.00			
				Average($256)					1.33	(0.67,2.64)		
				Poor($98)					2.49	(1.17,5.28)	<0.05	
	**Tsai**	**2005**	Perceived income adequacy						0.44	(0.32,0.61)	<0.01	
	**Adams**	**2004**	Facility: low income vs. full pay		-1.212	0.797			0.30	0.06-1.41	0.130	
	**Hays**	**1998**	Family income(<$6000)		0.13				1.14		<0.05	
	**Economic status**											
	**Wu**	**2020**	Economic status		0.01	0.56		0.03	1.01	0.34-3.03	0.981	
	**Gong**	**2012**	Self-reported family economic status (Ref. Good)	Bad					2.38	(1.08,5.25)	<0.05	
				Fair					1.08	(0.48,2.44)		
	**Economic stress**											
	**Kim**	**2012**	Economic stress			0.06	0.26				<0.001	
	**Chi**	**2005**	Self-rated financial strain						3.761	(2.328,6.081)	0.000	
	**Ability to meet costs**											
	**Chan**	**2011**	Ability to meet living	very low/low					3.30	(2.25,4.84)	<0.001	

			costs	Average/High/very high					1			
	**Chan**	**2009**	Ability to meet living costs	very low/low					2.58	(1.66,4.00)	<0.001	
				Average/High/very high					1			
**Health insurance**	**Hu**	**2018**	Medical insurance and welfare	Urban					1.205	(0.759,1.912)		
				Rural					0.714	(0.552,0.925)	<0.05	
				All					0.865	(0.694,1.076)		
	**Li**	**2016**	No health insurance		0.111	0.318			1.12	0.60-2.08		
**Pension**	**Hu**	**2018**	Pension	Urban					0.685	(0.484,0.970)	<0.05	
				Rural					0.792	(0.669,0.938)	<0.01	
				All					0.773	(0.666,0.898)	<0.01	
	**Li**	**2016**	Pension benefits	Low	-1.039	0.276			0.35	0.21-0.61	<0.001	
				Medium	-0.354	0.271			0.70	0.41-1.19		
				High	-1.004	0.319			0.37	0.20-0.68	<0.01	
**Housing**	**Compete**	**2018**	Household size						1.01	(0.90,1.14)		
	**Ng**	**2014**	Housing type (Ref.4-/5-room)	3-room					1.10	(0.61,1.98)	0.755	
				2-room					3.06	(1.52,6.16)	0.002	
	**Chan**	**2010**	1-2 room public	Men	0.6	(0.3)			1.82	1.01-3.28	<0.05	
				Women	0.7	(0.3)			2.01	1.12-3.63	<0.05	
			3 room public	Men	0.4	(0.1)			1.49	1.23-1.81	<0.05	
				Women	0.6	(0.1)			1.82	1.50-2.22	<0.05	
**Education**	**Bui**	**2020**	College or higher		-0.428	0.251			0.65	0.40-1.07		
	**Jin**	**2020**	Years of schooling		-0.019				0.98		0.447	

	**Kim**	**2020**	Less than High school		0.043	0.018			1.04	1.01-1.08	<0.05	
			High school graduate		0.034	0.015			1.03	1.01-1.07	<0.05	
	**Lee**	**2020**	>=High school	Men					1			
				Women					1			
			Middle school	Men					1.73	1.28-2.36		
				Women					1.45	1.04-2.03		
			Elementary school	Men					1.95	1.48-2.57		
				Women					1.59	1.20-2.11		
			No education	Men					2.5	1.78-3.51		
				Women					2.06	1.55-2.74		
	**Reynolds**	**2020**	Education		-0.01				0.99			
	**Wu**	**2020**	Education		-1.04	0.74		1.4	0.35	0.08-1.51	0.164	
	**Kim**	**2019**	Education		-0.09	0.030	-0.11		0.91	0.86-0.97	<0.01	
	**Chao**	**2018**	Education	Mild					1.01	(0.98,1.03)		
				Moderate severe					1.00	(0.97,1.03)		
	**Compete**	**2018**	Education	Primary education completed or more					0.64	(0.37,1.10)		
	**Hu**	**2018**	Education Literate and elementary school	Urban					0.867	(0.593,1.268)		
				Rural					1.079	(0.938,1.240)		
				All					1.013	(0.890,1.152)		
			Education High school +	Urban					0.477	(0.317,0.719)	<0.001	
				Rural					0.803	(0.633,1.020)	<0.10	

				All					0.555	(0.462,0.666)	<0.001	
	**Kim**	**2017**	Education				-0.30	-3.33			<0.01	
	**Park**	**2017**	More than high school				-0.04					
	**Aung**	**2016**	Education		-0.40				0.67	(-0.82,0.01)	0.06	
	**Chen**	**2016**	Education (Ref. secondary school or above)	Primary school	-0.19	0.37	-0.03		0.83	0.40-1.71		
				No education	0.07	0.49	0.01		1.07	0.41-2.80		
	**Li**	**2016**	Education	Can read and write	0.077	0.222			1.08	0.70-1.67		
				Primary school	-0.398	0.236			0.67	0.42-1.07		
				Junior high or more	-1.102	0.281			0.33	0.19-0.58	<0.001	
	**Vanoh**	**2016**	Education level		-0.10				0.91	(0.87,0.95)	<0.001	
	**Yoo**	**2016**	Education	Education ≤6 years			-0.01				0.93	
				Education>6 years			-0.10				0.06	
	**Jinhui Li**	**2015**	Education				-0.02					
	**Ng**	**2014**	Highest education level (Ref. secondary and higher)	Primary and lower					1.29	(0.94,1.77)	0.112	
	**Chen**	**2012**	Education level (Ref. 5years or more)	5 years or less	1.012	0.329			0.36	(0.21,0.79)	0.002	
	**Gong**	**2012**	Years of schooling (Ref. Low-0 year)	High(7+years)					0.81	(0.43,1.50)		
				Medium(1-6 years)					0.98	(0.67,1.44)		
	**Kim**	**2012**	Education level			0.49	-1.15				0.021	

	**Chao**	**2011**	Education		-0.007	(0.001)			0.993	0.991-0.995	<0.001	
	**Chan**	**2010**	Primary	Men		(0.2)	-0.2					
				Women		(0.2)	-0.2					
			Secondary	Men		(0.2)	-0.8				<0.05	
				Women		(0.2)	0.01					
			Vocational/Junior College/Polytechnic/university	Men		(0.2)	-1.1				<0.05	
				Women		(0.3)	-0.3					
	**Chi**	**2005**	Attained high school education						1.223	(0.640,2.335)	0.541	
	**Lee**	**2005**	≥Middle school	Anyang					1.14	(0.64,2.04)		
				Yoita					0.72	(0.48,1.09)		
				Total					0.84	(0.60,1.17)		
	**Chi**	**2001**	Years of education		-0.191	0.070	-0.082		0.83	0.72-0.95	<0.01	
	**Hays**	**1998**	Years of education		-0.018				0.98		<0.05	
**Area**	**Kim**	**2019**	Urban		-0.18	0.224	-0.02		0.84	0.54-1.30		
	**Chao**	**2018**	Years in the United States	Mild					1.00	(0.99,1.02)		
				Moderate severe					1.00	(0.99,1.02)		
			Years in community	Mild					0.98	(0.97,1.00)	<0.05	
				Moderate severe					1.00	(0.98,1.01)		
			Country of origin	Mild					1.12	(0.71,1.79_		
				Moderate severe					0.88	(0.52,1.47)		
	**Hu**	**2018**	Area Central China	Urban					1.418	(1.023,1.966)	<0.05	
				Rural					1.465	(1.254,1.710)	<0.001	

				All					1.391	(1.211,1.597)	<0.001	
			Area West China	Urban					1.845	(1.317,2.585)	<0.001	
				Rural					1.715	(1.471,1.999)	<0.001	
				All					1.690	(1.474,1.939)	<0.001	
	**Park**	**2017**	Length of stay in the USA				-0.02					
	**Li**	**2016**	Area	Rural	0.552	0.294			1.74	0.98-3.09		
	**Tahiri**	**2009**	Urban versus Metropolitan	Total population					1.75	(1.25,2.45)		
			Rural versus Metropolitan	Total population					2.01	(1.59,2.68)		
	**Leung**	**2007**	Location			0.621						-1.156
	**Chi**	**2005**	Lived in Hong Kong <20 years						2.378	(1.062,5.320)	0.035	
**Life event**	**Hays**	**1998**	Negative Life events		0.28				1.32		<0.01	
	**Gong**	**2012**	Family-related negative life events (Ref. none)	2 events and more					11.70	(7.72,17.73)	<0.01	
				1 event					4.01	(2.73,5.88)	<0.01	
	**Chen**	**2005**	Adverse Life events occurring in the Past 2 years Anything else severely up setting	No					1.00			
				Yes					3.54	(2.19,5.73)	<0.001	
			Adverse Life events	No					1.00			

			occurring in the Past 2 years<br>Horrifying experience, including accident, fire, physical attack, etc.	Yes					3.31	(1.70,6.43)	<0.001	
	**Yoo**	**2016**	Number of stressful life events				0.18				<0.01	
**Emotional state**	**Religious**											
	**Kim**	**2020**	Religiosity		0.003	0.003			1.00	1.00-1.01		
	**Wu**	**2020**	Religious preference		-1.23	0.73		1.68	0.29	0.07-1.22	0.095	
	**Chi**	**2005**	Having religious belief						0.993	(0.574,1.719)	0.981	
	**Adams**	**2005**	Church attendance/month		-0.382	0.117			0.68	0.54-0.86	0.001	
	**Loneliness**											
	**Mulat**	**2021**	Feeling of loneliness	Yes					1	0.62-1.61	0.972	
				No					1			
	**Vandoh**	**2016**	Loneliness		-0.00				1.00	(0.88,1.14)	0.971	
	**Jinhui Li**	**2015**	Loneliness (ULS-8)				0.41				<0.001	
	**Adams**	**2004**	UCLA Loneliness Scale		0.205	0.039			1.23	1.14-1.33	<0.0005	
**Health habits**	**Exercise**											
	**Wu**	**2020**	Regular exercise		-0.51	0.7		0.73	0.60	0.15-2.37	0.467	
	**Vandoh**	**2016**	Exercise		0.17				1.19	(0.90,1.56)	0.217	
	**Chi**	**2001**	physical performance		0.309	0.073	0.158		1.36	1.18-1.57	<0.0001	
	**Sleep**											
	**Mulat**	**2021**	Sleep medication	Yes					0.28	0.06-1.17	0.083	

				No					1			
	**Wu**	**2020**	Sleep quality		0.18	0.67		0.27	1.20	0.32-4.45	0.787	
			Nap habits		0.58	0.62		0.93	1.79	0.53-6.02	0.356	
	**Chan**	**2011**	Insomnia	No					0.20	(0.11,0.36)	<0.001	
				Yes					1			
	**Lee**	**2005**	Sleep <6 or >9 hours/day	Anyang					1.42	(0.87,2.33)		
				Yoita					1.48	(1.04,2.12)	<0.05	
				Total					1.45	(1.10,1.91)	<0.05	
	**Smoking**											
	**Mulat**	**2021**	Ever used tobacco	Yes					0.39	0.12-1.27	0.12	
				No					1			
	**Ahmad**	**2020**	Current smoker	Yes					4.19	1.69-10.39	0.002	
				No					R			
**Disease**	**Cognitive impairment**											
	**Adams**	**2020**	History of cognitive impairment	No					1			
				Yes					1.66	1.16-2.38	0.01	
	**Jin**	**2020**	Cognitive impairment		-0.032				0.97		0.153	
	**Kim**	**2017**	Cognitive function				-0.32	-3.37			<0.01	
	**Aung**	**2016**	Cognitive decline	Long-term memory loss	-0.52				0.59	(-0.93,-0.12)	0.01	
				Short-term memory loss	-0.62				0.54	(-1.16,0.07)	0.03	
	**Leung**	**2007**	Cognitive(impaired)			3.227					<0.01	8.602
	**Lee**	**2005**	Cognitive impairment	Anyang					4.06	(2.23,7.39)	<0.05	
				Yoita					1.60	(0.88,2.91)		

				Total					2.32	(1.56,3.43)	<0.05	
	**Tsai**	**2005**	Cognitive status						0.95	(0.90,1.00)	<0.05	
	**Hays**	**1998**	Cognitive impairment		0.14				1.15			
	**Chronic disease**											
	**Mulat**	**2021**	Known chronic disease	Yes					1.91	1.30-2.81	0.001	
				No					1			
	**Ahmad**	**2020**	Chronic medical illness	Yes					1.44	1.15-1.81	0.002	
				No					R			
	**Jin**	**2020**	Number of chronic diseases		0.08				1.08		<0.001	
	**Hu**	**2018**	chronic disease	Urban					2.256	(1.537,3.312)	<0.001	
				Rural					2.212	(1.911,2.562)	<0.001	
				All					2.175	(1.898,2.493)	<0.001	
	**Park**	**2017**	Chronic conditions				0.1					
	**Li**	**2016**	Chronic conditions	One	0.908	0.208			2.48	1.65-3.73	<0.001	
				Two	1.692	0.239			5.43	3.40-8.67	<0.001	
				Three or more	2.495	0.257			12.12	7.32-20.06	<0.001	
	**Yoo**	**2016**	No. of chronic diseases				0.18				<0.01	
	**Chan**	**2010**	Number of chronic diseases	Men	0.1	(0.04)			1.11	1.02-1.20	<0.05	
				Women	0.5	(0.04)			1.65	1.52-1.78	<0.05	
	**Tahiri**	**2009**	Chronic condition (Each additional unit)	Metropolitan					1.37	(1.07,1.77)		
				Urban					1.30	(0.94,1.80)		

				Rural					1.30	(1.04,1.61)		
				Total population					1.32	(1.14,1.52)		
	**Leung**	**2007**	Diseases(Yes)			2.231					<0.05	5.406
	**Adams**	**2004**	Number of chronic health conditions		0.945	0.250			2.57	1.58-4.20	<0.0005	
	**Hays**	**1998**	Chronic Health problems		0.06				1.06		<0.05	
	**Hypertension**											
	**Kim**	**2019**	Hypertension		-0.21	0.200	-0.03		0.81	0.55-1.20		
	**Vandoh**	**2016**	Hypertension		0.28				1.32	(1.02-1.71)	0.034	
	**Kim**	**2012**	Hypertension			0.50	0.99				0.052	
	**Chan**	**2009**	Type of chronic illness: hypertension	Yes					1.80	(1.15,2.81)	0.010	
				No					1			
	**Chen**	**2005**	Hypertension	No					1.00			
				Yes, detected					0.85	(0.44,1.66)		
				Yes, undetected					1.78	(1.05,3.01)	<0.05	
	**Diabetes**											
	**Kim**	**2019**	Diabetes		-0.34	0.235	-0.04		0.71	0.45-1.13		
	**Wee**	**2014**	Diabetes						1.72	(0.97,3.03)	0.062	
	**Fall**											
	**Chen**	**2016**	Recent fall history		0.73	0.38	0.09		2.08	0.99-4.37		
	**Wee**	**2014**	Falls						2.72	(1.59,4.67)	<0.001	
	**Visual**											
	**Wee**	**2014**	Visual impairment						2.37	(1.28,4.39)	0.006	
	**Chi**	**2005**	Vision problem						2.052	(1.214,3.470)	0.007	

	**Neuroticism**											
	**Vandoh**	**2016**	Neuroticism		0.09				1.10	(1.03,1.14)	<0.001	
	**Handerson**	**1997**	Neuroticism, Wave 1				0.077				0.056	
	**Stroke**											
	**Chan stroke**	**2011**	Type of chronic illness: stroke	No					0.39	(0.16,0.95)	0.039	
				Yes					1			
	**Kim**	**2019**	Stroke		0.43	0.543	0.03		1.54	0.53-4.46		
	**Comorbidity**											
	**Wu**	**2020**	Comorbidity		-0.52	0.98		0.53	0.59	0.09-4.06	0.596	
	**Chao**	**2018**	Medical comorbidities	Mild					1.22	(1.13,1.31)	<0.001	
				Moderate severe					1.25	(1.14,1.37)	<0.001	
	**Compte**	**2018**	no Self-reported comorbidities						1.21	(1.06,1.39)	<0.001	
**Ability**	**ADL/IADL**											
	**Ahmad**	**2020**	Limitation in ADL	Yes					2.58	2.01-3.32	<0.001	
				No					R			
			Limitation in IADL	Yes					1.68	1.32-2.14	<0.001	
				No					R			
	**Jin**	**2020**	ADL score		-0.053				0.95		0.031	
			IADL score		-0.067				0.94		0.016	
	**Wu**	**2020**	Barthel index		-0.02	0.03		0.67	0.98	0.92-1.04	0.504	
			IADL score		-0.05	0.26		0.2	0.95	0.57-1.58	0.843	
	**Gu**	**2019**	Functional ability (ADL)	Mild impairment vs. normal functional ability	1.20	0.50			3.31	(1.25,8.75)	0.02	

				Severe impairment vs. normal functional ability	1.55	0.59			4.72	(1.50,14.84)	0.01	
	**Kim**	**2019**	IADL dependent		0.69	0.276	0.09			1.16-3.42	<0.05	
	**Compte**	**2018**	Functional impairment	ADL impairment					1.95	(1.20,3.17)	<0.001	
				IADL impairment					1.15	(0.68,1.95)		
	**Hu**	**2018**	ADLs	Urban					0.941	(0.855,1.036)		
				Rural					1.085	(1.041,1.132)	<0.001	
				All					1.059	(1.020,1.099)	<0.01	
			IADLs	Urban					1.184	(1.117,1.256)	<0.001	
				Rural					1.142	(1.111,1.173)	<0.001	
				All					1.148	(1.121,1.177)	<0.001	
	**Park**	**2017**	Functional disability (ADL, IADL)				0.04					
	**Chen**	**2016**	ADLs		-0.07	0.01	-0.27		0.93	0.91-0.95	<0.001	
	**Li**	**2016**	ADL disability		2.145	0.231			8.54	5.43-13.43	<0.001	
			IADL disability		2.295	0.225			9.92	6.39-13.43	<0.001	
	**Vadoh**	**2016**	IADL score		-0.09				0.92	(0.87,0.98)	0.005	
	**Chen**	**2012**	IADL (Ref. non-impaired)	Impaired	0.851	0.308			2.34	(1.28,4.28)	0.006	
	**Chen**	**2010**	At least one ADL limitation	Men	0.6	(0.4)			1.82	0.83-3.99		
				Women	0.9	(0.3)			2.46	1.37-4.43	<0.05	
			At least one IADL	Men	2.0	(0.4)			7.39	3.37-16.18	<0.05	

			limitation	Women	0.3	(0.3)			1.35	0.75-2.43		
	**Leung**	**2007**	ADL			0.101					0.043	
	**Chi**	**2005**	Severity of ADL impairment						1.213	(1.045,1.408)	0.011	
	**Chi**	**2001**	ADL		-0.012	0.237	-0.002		0.99	0.62-1.57		
			IADL		0.317	0.137	0.194		1.37	1.05-1.80	<0.05	
	**Handerson**	**1997**	ADL, Wave 1				-0.103				0.033	
			ADL, Wave 2				0.283				0.012	
			ADL squared, Wave 2				-0.150				0.076	
	**Functional disability**											
	**Kim**	**2020**	Functional limitations		0.233	0.039			1.26	1.17-1.36	<0.001	
	**Reynold**	**2020**	W1 Functional health problems		0.1				1.11		<0.001	
	**Yoo**	**2016**	Functional independence				-0.16				<0.01	
	**Lee**	**2005**	Functional capacity	Anyang					0.92	(0.86,0.99)	<0.05	
				Yoita					0.88	(0.82,0.94)	<0.05	
				Total					0.90	(0.86,0.94)	<0.05	
	**Hays**	**1998**	Functional disability		0.21				1.23		<0.001	
	**Physical disability**											
	**Chi**	**2001**	Physical disability	Yes					1.86	0.62-5.53	0.263	
				No					1			
	**Jin**	**2020**		Physical frailty score	0.273				1.31		<0.001	
	**Chao**	**2011**	Physical status		0.304	(0.011)			1.36	1.33-1.38	<0.001	
	**Following task**											

	**Vanoh**	**2016**	Poor fitness (score from chair sit and reach test)		0.03				1.03	(1.02,1.04)	<0.001	
	**Chan**	**2009**	Ability to the following tasks Able to do heavy housework	No					3.99	(1.85,8.57)	<0.001	
				Yes					1			
**Perceived health**	**Wu**	**2020**	Perceived health		2.71	0.84		3.21	15.03	2.90-77.98	0.002	
	**Kim**	**2019**	Self-rated health		-1.56	0.142	-0.42		0.21	0.16-0.28	<0.001	
	**Compte**	**2018**	Self-reported health status	Poor/very poor					1.85	(0.83,4.10)		
				Fair					1.16	(0.68,1.96)		
	**Aung**	**2016**	Self-impression of health		-1.03				0.36	(-1.58,-0.49)	<0.001	
	**Yoo**	**2016**	Current health status	Poor subjective health			0.30				<0.01	
				Good subjective health			-0.14				0.01	
	**Gong**	**2012**	Self-perceived physical health (Ref. Good)	Bad					4.74	(2.72,8.28)	<0.01	
				Fair					1.61	(0.88,2.93)		
	**Kim**	**2012**	Perceived health status			0.30	-0.61				0.043	
	**Chan**	**2011**	Perceived health status	very poor/poor					2.47	(1.42,4.30)	0.001	
				Average/good/very good					1			
	**Chan**	**2009**	Perceived health	very poor/poor					4.15	(2.65,6.49)	<0.001	
				Average/good/very good					1			

	**Tahiri**	**2009**	Self-rated health (One additional point poor health)	Metropolitan					1.15	(0.94,1.42)		
				Urban					1.14	(0.85,1.53)		
				Rural					1.24	(1.02,1.51)		
				Total population					1.19	(1.05,1.35)		
	**Shin**	**2008**	Poor health						1.81	(0.88,3.80)	0.11	
	**Chen**	**2005**	Self-assessed physical health status	Very good or good					1.00			
				Average					2.49	(1.19,5.22)	<0.05	
				Poor					3.11	(1.45,6.70)	<0.01	
	**Chi**	**2005**	Poor self-rated health status						1.780	(1.092,2.898)	0.020	
	**Lee**	**2005**	Poor self-rated health	Anyang					6.34	(3.61,11.14)	<0.05	
				Yoita					2.30	(1.58,3.33)	<0.05	
				Total					3.08	(2.30,4.12)	<0.05	
	**Tsai**	**2005**	Perceived health status						0.56	(0.46,0.70)	<0.01	
**Pain**	**Jin**	**2020**	Pain		0.096				1.10		<0.001	
	**Chi**	**2005**	Subjective long-term pain						1.925	(1.175,3.155)	0.009	
**Hospital**	**Li**	**2016**	No physician visit when ill		1.998	0.296			7.37	4.13-13.17	<0.001	
			No hospitalization when needed		2.369	0.409			10.69	4.79-23.82	<0.001	
			Early self-discharge from hospital		0.756	0.429			2.13	0.92-4.94		
	**Lee**	**2005**	Hospitalization	Anyang					0.84	(0.48,1.47)		
				Yoita					0.77	(0.44,1.35)		
				Total					0.83	(0.57,1.21)		

Table S2. various depression intervention methods.

	Author	Year	Measurement	Variable		Coefficient(B)	Standard Error(SE)	Standardized Coefficient(β)	t	OR	95%CI	p
Social support	Mulat	2021	The Oslo-3 Social Support Scale (OSS-3)	Perceived social support	poor					3.32	1.77-6.23	<0.001
					Moderate					1.24	0.66-2.34	0.498
					Strong					1		
	Adams	2020	The Oslo-3 Social Support Scale (OSS-3)	Social support availability	poor					1		
					Moderate					0.56	0.38-0.82	0.003
					Strong					0.27	0.17-0.44	<0.001
	Compete	2018	The Oslo-3 Social Support Scale (OSS-3)	Social support						0.32	(0.19,0.54)	**<0.001**
	Ahmad	2020	Duke's Social Support Index	Social support	good					R		
					poor					4.3	2.98-6.20	<0.001
	Jinhui Li	2015	Social support (DSSI-10; Duke social support index)	Social support (DSSI-10)				-0.18				<0.05
	Bal	2020	The framework of the World Bank's Social Capital Assessment Tool and previous works of our research group : six dimensions of social capital	social support		0.18	0.05			1.20	1.08-1.32	0.001
	Bui	2020	Social support scale	Perceived support		-0.219	0.054			0.80	0.72-0.89	<0.001
	Jin	2020	Social Support Rating	Objective support		-0.015				0.99		0.511

			Scale (SSRS)	Subjective support		-0.188				0.83		<0.001
				Support utilization		-0.08				0.92		<0.001
	Lee	2020	Emotional support exchange	Reciprocity	Men					1		
					Women					1		
				Giving or receiving only	Men					1.77	1.25-2.52	
					Women					1.64	1.32-2.04	
				No	Men					2.08	1.53-2.83	
					Women					2.19	1.79-2.68	
	Wu	2020	Chinese version of the Multidimensional Scale of Perceived Social Support	Social support		-0.13	0.04		2.94	0.88	0.81-0.95	0.004
	Chao	2018	Positive Perceived Social Support (Positive PSS)	Positive Perceived social support	Mild					0.88	(0.85,0.92)	**<0.001**
					Moderate severe					0.82	(0.79,0.86)	**<0.001**
	Gayman	2018	Perceived social support (a modified and shortened version of the Provisions of Social Relations scale) 1)Family support(16items) 2)Friend support(8items)	Perceived social support	Family support		0.91	-0.29				<0.001
	Hu	2018	Family support	Living with a spouse	Urban					0.678	(0.491,0.937)	**<0.05**
					Rural					0.814	(0.704,0.940)	**<0.01**
					All					0.777	(0.682,0.885)	**<0.001**

				Number of Children and grandchildren	Urban					0.915	(0.848,0.988)	**<0.05**
					Rural					0.943	(0.916,0.971)	**<0.001**
					All					0.952	(0.927,0.978)	**<0.001**
				Frequency of contacting with children	Urban					0.986	(0.938,1.035)	
					Rural					0.958	(0.937,0.980)	**<0.001**
					All					0.955	(0.936,0.974)	**<0.001**
			Community support	Senior center in community	Urban					0.634	(0.467,0.861)	**<0.01**
					Rural					0.684	(0.580,0.807)	**<0.001**
					All					0.636	(0.551,0.734)	**<0.001**
				Elderly association	Urban					0.962	(0.705,1.312)	
					Rural					0.804	(0.671,0.963)	**<0.05**
					All					0.802	(0.689,0.934)	**<0.01**
				Organizing activities frequently	Urban					0.544	(0.419,0.706)	**<0.001**
					Rural					0.964	(0.853,1.089)	
					All					0.851	(0.763,0.951)	**<0.01**
			Public support	Pension	Urban					0.685	(0.484,0.970)	**<0.05**
					Rural					0.792	(0.669,0.938)	**<0.01**
					All					0.773	(0.666,0.898)	**<0.01**
				Medical insurance and welfare	Urban					1.205	(0.759,1.912)	
					Rural					0.714	(0.552,0.925)	**<0.05**
					All					0.865	(0.694,1.076)	

	Kim	2017	Social support (MOSS-E; The Measurement Of Social Support in the Elderly scale)	Social support					-0.31	-3.26			<0.01
	Ang	2016	Received social support	Received social support	Male			0.07	-0.01				
					Female			0.07	-0.29				<0.001
	Li	2016	Social support and participation 1)partnered status 2)children nearby 3)social participation 4)elderly activity center in community	Social support and participation	Partnered		-0.738	0.2			0.48	0.32-0.71	<0.001
					Children nearby		-0.032	0.2			0.97	0.65-1.43	
					Social participation		**-1.240**	0.174			0.29	0.21-0.41	<0.001
					Elder activity center		-0.215	0.24			0.81	0.50-1.29	
	Tusboi	2016	Receiving emotional support (RES)	from partner	Married	Men			-0.041			(-0.43,-0.10)	<0.005
						Women			-0.068			(-0.55,-0.21)	<0.0005
				from children	Married	Men			0.045			(0.12,0.41)	<0.0005
						Women			0.018			(-0.06,0.24)	
					Single	Men			0.028			(-0.27,0.60)	
						Women			-0.028			(-0.35,0.04)	
				from outside family	Married	Men			-0.014			(-0.24,0.07)	
						Women			0.014			(-0.09,0.23)	
					Single	Men			-0.006			(-0.51,0.44)	
						Women			0.028			(-0.05,0.35)	
			Giving emotional support (GES)	to partner	Married	Men			-0.038			(-0.39,-0.09)	<0.005
						Women			-0.033			(-0.33,-0.03)	<0.05
				to children	Married	Men			**-0.040**			(-0.36,-0.08)	<0.005
						Women			0.005			(-0.13,0.18)	
					Single	Men			-0.023			(-0.57,0.30)	

						Women			-0.043			(-0.42,-0.05)	<0.05
				to outside family	Married	Men			-0.074			(-0.57,-0.27)	<0.0005
						Women			-0.093			(-0.65,-0.32)	<0.0005
					Single	Men			-0.112			(-1.14,-0.21)	<0.005
						Women			-0.119			(-0.86,-0.45)	<0.0005
			Receiving instrumental support (RIS)	from partner	Married	Men			-0.024			(-0.47,-0.02)	<0.05
						Women			-0.058			(-0.54,-0.19)	<0.0005
				from children	Married	Men			-0.041			(-0.36,-0.09)	<0.005
						Women			-0.044			(-0.38,-0.08)	<0.005
					Single	Men			-0.129			(-1.29,-0.39)	<0.0005
						Women			-0.037			(-0.49,-0.03)	<0.05
				from outside family	Married	Men			**0.030**			(0.15,0.84)	<0.005
						Women			-0.015			(-0.42,0.11)	
					Single	Men			**0.040**			(-0.31,1.07)	
						Women			-0.015			(-0.39,0.15)	
			Giving instrumental support (GIS)	to partner	Married	Men			-0.051			(-0.76,-0.32)	<0.0005
						Women			**-0.040**			(-0.57,-0.13)	<0.005
				to children	Married	Men			-0.011			(-0.19,0,07)	
						Women			-0.031			(-0.31,-0.01)	<0.05
					Single	Men			-0.077			(-0.83,-0.06)	<0.05
						Women			-0.096			(-0.74,-0.37)	<0.0005
				to outside family	Married	Men			-0.033			(-0.53,-0.10)	<0.005
						Women			**-0.020**			(-0.33,0.05)	
					Single	Men			**-0.030**			(-0.85,0.36)	
						Women			-0.075			(-0.73,-0.27)	<0.0005
	Vanoh	2016	Medical Outcome study Social Support (MOSS)	Social support			-0.01				0.99	(0.99,1.00)	0.222

	Yoo	2016	Social support (PSSS; The Perceived Social Support Scale)	Social support				-0.11				0.04
	Ng	2014	Social support 1)Living arrangement 2)Frequency of leisure time spent 3)Social isolation	Living arrangement (Ref. Spouse with child(ren) /grandchild(ren))	Spouse only					1.05	(0.57,1.93)	0.879
					Child(ren)/grandchild(ren) only					0.74	(0.31,1.77)	0.494
					Alone or with domestic helper					2.73	(1.31,5.69)	0.007
					Others (relatives, friends, tenant)					1.82	(0.78,4.24)	0.166
				Frequency of leisure time spent (Ref. At least once a month)	Less than once a month					1.51	(1.04,2.19)	0.028
					Childless					1.73	(1.14,2.60)	0.009
				Social isolation (Never of rarely)	Occasionally or often					7.12	(4.87,10.40)	<0.001
	Gong	2012	Support from family members	Support from family members (Ref. Good)	Bad					6.93	(3.26,14.70)	<0.01
					Fair					2.90	(1.52,5.53)	<0.01
	Kim	2012	1) Family & Friend support (12 items) 2)Social support(20items)	Family support	men	-0.23	0.11			0.79	0.64-0.99	0.044
				Family support	women	-0.18	0.09			0.84	0.70-0.99	0.038
				friend support	women	-0.28	0.09			0.76	0.63-0.90	0.002
	Wang	2012	Multidimensional Scale of Perceived Social Support	Social support	Family support	**0.243**				1.28		**0.180**
					Friend support	**0.229**				0.00		**0.002**

			(MSPSS) 1)Family support 2)Friend support 3)Other support		Other support	**0.049**				1.05		**0.778**
	Chao	2011	Social support	Social network size	With spouse	-0.047	(0.013)			0.95	0.93-0.98	<0.001
				Social network size	No. of children	0.001	(0.002)			1.00	1.00-1.004	
				Social network size	No. of relatives	-0.032	(0.007)			0.97	0.96-0.98	<0.001
				Social network size	No. of friends	-0.017	(0.007)			0.98	0.97-0.996	<0.01
				Social network composition (Ref: family members accounted for 100% of the network)	Family<24.99%	0.044	(0.044)			1.04	0.96-1.14	
					Family=25-49.99%	0.027	(0.040)			1.03	0.95-1.11	
					Family=50-99.99%	**0.040**	(0.036)			1.04	0.97-1.12	
				Frequency of social contact		**-0.013**	(0.006)			0.99	0.98-0.998	<0.05
				Proximity of support		**-0.076**	(0.011)			0.93	0.91-0.95	<0.001
				Types of support received	Instrumental support (Ref: with needs and received instrumental support): No assist	**0.131**	(0.017)			1.14	1.10-1.18	<0.001
					Instrumental support (Ref: with needs and received instrumental support): No need	**-0.007**	(0.010)			0.99	0.97-1.01	
					Emotional support	**-0.030**	(0.004)			0.97	0.96-0.98	<0.001
					Financial support	**-0.003**	(0.011)			1.00	0.98-1.02	

					Financial support	**-0.029**	(0.014)			0.97	0.95-0.998	<0.05
				Helping others	Housework	**-0.026**	(0.013)			0.97	0.95-0.999	<0.05
					Physical care	**0.079**	(0.023)			1.08	1.03-1.13	<0.001
				Satisfaction with social support		**-0.177**	0.006			0.84	0.83-0.85	<0.001
	suttajit	2010	The scale of Six Social Support deficits	Living alone without a child or other relative						0.7	(0.4,1.1)	
				Seeing a child or other relative less often than once per week						1.3	(0.5,3.1)	
				Lack of reciprocity with neighbors						1.9	(1.4,2.5)	
				Lack of reciprocity between children and extended family members						2.6	(1.9,3.6)	
				Difficulty in relationship with one or more relatives						2.3	(1.4,3.7)	
				Dissatisfaction with support from children						1.9	(1.2,3.0)	
				Number of social support deficits	0					1.0	(Ref.)	
					1					1.7	(1.2,2.4)	
					2					3.0	(2.0,4.4)	
					≥3					4.0	(2.3,6.9)	
	Tahiri	2009	Functional relationship (social support, presence of conflict)	Social support score	Metropolitan					0.49	(0.30,0.81)	
					Urban					1.10	(0.48,2.48)	
					Rural					0.66	(0.41,1.07)	
					Total population					0.64	(0.47,0.88)	
				No conflict versus no children	Metropolitan					0.74	(0.43,1.28)	
					Urban					1.00	(0.43,2.34)	
					Rural					0.90	(0.52,1.57)	
					Total population					0.88	(0.62,1.25)	
				Conflict versus no	Metropolitan					1.14	(0.64,2.03)	

				children	Urban					1.77	(0.69,4.54)	
					Rural					1.29	(0.69,2.40)	
					Total population					1.36	(0.93,1.99)	
				No conflict versus no spouse	Metropolitan					0.56	(0.21,1.45)	
					Urban					0.29	(0.10,0.93)	
					Rural					0.46	(0.22,0.93)	
					Total population					0.45	(0.27,0.75)	
				Conflict versus no spouse	Metropolitan					0.90	(0.56,1.43)	
					Urban					0.70	(0.37,1.34)	
					Rural					0.71	(0.46,1.07)	
					Total population					0.76	(0.58,1.01)	
	Shin	2008	Medical Outcome Study Social Support Survey (MOS-SSS)	Poor social support						3.05	(1.77,5.27)	<0.001
	Leung	2007	Social Support Rating Scale(SSRS) Chinese modification of the Family Emotional Involvement and Criticism Scale(FEICS)	Instrumental support		-0.003	0.083			1.00	0.84-1.17	
				Emotional support		-0.227	0.084			0.80	0.68-0.94	<0.01
	Chen	2005	Social support 1)Quality 2)Quantity 3)Community participation	Good relationships with neighbors	Yes					1.00		
					No					1.66	(1.04,2.66)	<0.05
				Living with spouse, children, or grandchildren						1.00		
				Living with parents						4.31	(1.27,14.70)	<0.05
				Living with no one						1.98	(1.15,3.42)	<0.05
	Koizum	2005	Social support	To consult in trouble						2.6	(1.2,5.3)	<0.05

	i		questionnaire	To consult in bad physical condition						1.7	(0.8,3.7)	
				To help with your daily housework						1.1	(0.6,2.3)	
				To take to a hospital						1.8	(0.9,3.8)	
				To take care of you						3.0	(1.4,6.1)	<0.05
	Lee	2005	Social support index: Comprised of both receiving and giving social support	Social support	Anyang					0.85	(0.79,0.91)	<0.05
					Yoita					0.94	(0.89,0.99)	<0.05
					Total					0.90	(0.86,0.94)	<0.05
	Chi	2001	social support	Network size	Marital status	-1.343	0.65	-0.067		0.26	0.07-0.93	<0.05
					Number of relatives	0.025	**0.040**	0.023		1.03	0.95-1.11	
					Number of relatives seen once a month	-0.057	0.054	0.047		0.94	0.85-1.05	
					Number of relatives felt close to	**-0.210**	0.073	**-0.110**		0.81	0.70-0.94	<0.01
					Number of friends seen once a month	-0.234	0.139	-0.101		0.79	0.60-1.04	
					Number of friends felt close to	0.128	0.153	**0.050**		1.14	0.84-1.53	
				Network composition	Network composition of relatives and friends felt close to	-0.405	0.374	**-0.050**		0.67	0.32-1.39	
					Network composition of relatives and friends seen once a month	0.894	0.419	0.093		2.44	1.08-5.56	<0.05

				Social contact frequency	Frequency of contact with relatives	-0.549	0.183	-0.087		0.58	0.40-0.83	<0.01
					Frequency of contact with friends	-0.283	0.181	-0.055		0.75	0.53-1.07	
					Satisfaction with social support	-1.777	0.389	-0.146		0.17	0.08-0.36	<0.0001
				Instrumental and emotional support	Frequency of discussion about decisions	-0.285	0.242	-0.043		0.75	0.47-1.21	
					Willing to listen to respondents' problems	-0.011	**0.330**	**0.000**		0.99	0.52-1.89	
					Reliable in giving care when ill	-0.719	0.317	-0.076		0.49	0.26-0.91	<0.05
					Tangible help from relatives living with respondent	-0.239	0.133	**-0.060**		0.79	0.61-1.02	
					Tangible help from relatives not living with respondent	**-0.470**	0.125	-0.118		0.63	0.49-0.80	<0.01
				Helping others	Willing to discuss others' problems	0.344	0.254	**0.050**		1.41	0.86-2.32	
					Frequency of helping others	-0.013	0.166	-0.002		0.99	0.71-1.37	
					Frequency of providing comfort	-0.009	0.274	-0.001		0.99	0.58-1.70	
					Frequency of providing financial help	-0.434	0.241	-0.053		0.65	0.40-1.04	

	Hays	1998	Perceived social support	Availability of a confidant		-0.14				0.87		<0.001
				satisfaction with amount of social interaction		-0.17				0.84		<0.001
				Instrumental support Received		0.028				1.03		<0.01
				Instrumental support Given		-0.003				1.00		
				Size of Social Network		-0.015				0.99		<0.001
	Antonucci	1997	social support variable	Social support variables	Satisfaction	-3.10	0.39			0.05	0.02-0.10	<0.001
					Understanding	-3.34	0.29			0.04	0.02-0.06	<0.001
	Henderson	1997	Social support	Social support/inactivity	Social support-friends Wave 2			-0.095				0.015
					Social support-social visits, Wave 2			-0.087				0.032
					Activity level, Wave 2			0.095				0.025
Social participation	Choi	2020	Social participation, Emotional social support : Additional survey of the Korean Retirement and Income Study(KReIS)	Economic activity - Yes	All					0.97	0.82-1.14	
					Female					0.87	0.70-1.08	
					Male					1.14	0.87-1.49	
				Economic activity - No	All					1		
					Female					1		
					Male					1		
				Social activity - Yes	All					0.60	0.52-0.70	<0.001
					Female					0.51	0.42-0.62	<0.001
					Male					0.81	0.63-1.04	
				Social activity - No	All					1		

					Female					**1**		
					Male					**1**		
				Volunteer work - Yes	All					0.42	0.24-0.74	<0.01
					Female					0.42	0.19-0.91	<0.01
					Male					0.42	0.18-0.96	<0.01
				Volunteer work - No	All					1		
					Female					**1**		
					Male					**1**		
				Donation - Yes	All					0.56	0.43-0.74	<0.001
					Female					0.73	0.51-1.04	
					Male					0.39	0.25-0.60	<0.001
				Donation - No	All					1		
					Female					1		
					Male					1		
	Bal	2020	The framework of the World Bank's Social Capital Assessment Tool and previous works of our research group : six dimensions of social capital	social participation		0.35	0.07			1.42	1.24-1.63	<0.001
	Lee	2020	Social participation	>=2	Men					1		
					Women					1		
				1	Men					2.06	1.41-3.01	
					Women					1.97	1.57-2.49	
				0	Men					4.38	3.01-6.38	

					Women					3.55	2.78-4.53	
	Yamaguchi	2019	Civic participation	community level	Men		0.011			0.93	(0.88,0.99)	<0.05
					Women		0.014			0.94	(0.88,0.997)	<0.05
				individual level	Men		0.013			0.75	(0.71,0.80)	<0.001
					Women		0.019			0.82	(0.78,0.87)	<0.001
	Li	2016	Social support and participation 1)partnered status 2)children nearby 3)social participation 4)elderly activity center in community	Social support and participation	Partnered	-0.738	0.2			0.48	0.32-0.71	<0.001
					Children nearby	-0.032	0.2			0.97	0.65-1.43	
					Social participation	**-1.240**	0.174			0.29	0.21-0.41	<0.001
					Elder activity center	-0.215	0.24			0.81	0.50-1.29	
	Tahiri	2009	Structural relationship (Informal network, Formal network)	Volunteer work :Yes versus no	Metropolitan					0.67	(0.42,1.05)	
					Urban					0.98	(0.53,1.79)	
					Rural					0.73	(0.50,1.07)	
					Total population					0.76	(0.59,0.99)	
Social connection/ socialnetwork	Gu	2019	Lubben social network scale (LSNS-6)	Social support network	Social isolation risks vs. normal Social support network	0.95	0.43			2.59	(1.13,5.96)	0.03
	Kim	2019	Lubben social network scale Revised (LSNS-R)	Friend support network		-0.58	0.325	-0.06		0.56	0.30-1.06	
				Family support network		-0.72	0.331	-0.07		0.49	0.25-0.93	<0.05
				Diverse support network		-1.11	0.277	-0.13		0.33	0.19-0.57	<0.001
	Park	2017	Social network (LSNS-6; Lubben social network scale)	Social network				-0.23				<0.01

	Wee	2014	Social network (LSNS-6; Lubben Social Network Scale)	Social network (LSNS-6score>12)							0.27	(0.14,0.51)	<0.001
	Chen	2012	family support 1)Family social support 2)Living status (LSNS; Lubben Social Network Scale)	Living status (Ref. with someone)	Alone or with domestic helper		0.551	0.373			**1.73**	(0.84,3.60)	0.035
	Chan	2011	Lubben Social Network Scale (SNS)	social network scale	19 or below(not enough)						2.2	(1.26,3.83)	0.005
					20+(enough/good)						1		
	Chan	2010	Living arrangement Modified Lubben's revised social network scale(LSLS-12)	Living arrangements	Living alone	Men		(0.3)	1.2				<0.05
						Women		(0.2)	1.7				<0.05
					Living with at least one child, no spouse	Men		(0.2)	0.8				<0.05
						Women		(0.2)	0.4				<0.05
					Loving with a spouse, no child	Men		(0.1)	-0.3				<0.05
						Women		(0.2)	-0.03				
					Living	Men		(0.3)	0.3				

					only with others	Women		(0.3)	0.4				
				Social network outside the household	Weak	Men		(0.2)	1.3				<0.05
						Women		(0.2)	0.4				<0.05
	Chan	2009	Lubben Social Network Scale(LSNS)	Social network scale	19 or below(not enough)						3.63	(2.35,5.60)	<0.001
					20+ (enough/good)						1		
	Chi	2005	Lubben Social Network Scale(LSNS)	High social support(LSNS)							**0.935**	(0.906,0.965)	**0.000**
	bal	2020	The framework of the World Bank's Social Capital Assessment Tool and previous works of our research group : six dimensions of social capital	social connection			0.76	0.12			2.14	1.69-2.71	<0.001
	Bui	2020	Network structure Social network function	Network structure	Network size		0.037	0.09			1.04	0.87-1.24	
					Number living with ego		0.111	0.118			1.12	0.89-1.41	
					Proportion female		-0.365	0.428			0.69	0.30-1.61	
					Number of close		0.008	0.067			1.01	0.88-1.14	

					ties							
					Density	0.79	0.349			2.20	1.11-4.37	<0.05
					Frequency of contact with alters (contact-days per year)	-0.0003	0.0003			1.00	0.999-1.000	
	Kim	2020	1) Network size (Individual-level) 2)Social interaction(Individual-level)	Individual-level	Social interaction	-0.014	0.006			0.99	0.97-0.997	<0.05
					Network size	-0.008	0.004			0.99	0.98-0.999	<0.05
					Functional limitations	0.233	0.039			1.26	1.17-1.36	<0.001
				Contextual-level	Neighborhood fear	-0.005	0.022			1.00	0.95-1.04	
					Social cohesion	-0.133	0.053			0.88	0.79-0.97	<0.05
	Lee	2020	Social network	Social network	Men					0.8	0.76-0.86	
					Women					0.79	0.75-0.83	
	Reynolds	2020	community-layer connection interpersonal-layer connection partner-layer connection	Baseline partner connection		-0.05				0.95		<0.01
				Baseline Interpersonal connection		-0.02				0.98		
				Baseline Community connection		-0.09				0.91		<0.001
	Wu	2020	Chinese version of the Intergenerational Relationship Scale	Intergenerational relationships		-0.07	0.03		2.47	0.93	0.88-0.99	0.015
	Aung	2016	Social Network Index (SNI)	Social network	Social network index			-0.18			(-0.28,-0.09)	<0.001
	Chen	2016	Neighborhood support network 1)Family living	Support network	Family living together	-0.46	0.43	-0.07		0.63	0.27-1.47	
					Family and	0.13	0.35	0.02		1.14	0.57-2.26	

			together<br>2)Family and relatives<br>3)Friends<br>4)Organizations		relatives							
					Neighbors and friend	-1.10	0.55	-0.09		0.33	0.11-0.98	<0.05
					Organizations	1.17	0.51	0.11		3.22	1.19-8.75	<0.05
	Tsai	2005	Social support scale<br>1)Social support network<br>2)Quantities of social support<br>3)Satisfaction of social support	Social support network						0.88	(0.82,0.94)	<0.01
	Adams	2004	Number visitors/week<br>Visitor type	Number visitors/week		**0.117**	**0.140**			1.12	0.85-1.48	**0.406**
				visitor: Neighbor		**-1.275**	**0.643**			0.28	0.08-0.99	**0.049**
				visitor: Adult child		**0.268**	**0.614**			1.31	0.39-4.36	**0.663**
				visitor: Friend		**-0.606**	**0.647**			0.55	0.15-1.94	**0.350**
				Lubben social network scale		**-0.027**	**0.046**			0.97	0.89-1.07	**0.558**
	Antonucci	1997	Social relation : version of portions of the Social networks in Adult life Questionnaire	Social network variables	Size	-1.49	0.19			0.23	0.16-0.33	<0.001
				Composition(family, friends)	All family	0.48	0.41			1.62	0.72-3.61	
					Mostly family	0.15	0.42			1.16	0.51-2.65	
					Mostly friends	0.02	0.57			1.02	0.33-3.12	
					All friends	2.00	0.74			7.39	1.73-31.51	0.007
